# Seasonal drivers of understorey temperature buffering in temperate deciduous forests across Europe

**DOI:** 10.1111/geb.12991

**Published:** 2019-08-22

**Authors:** Florian Zellweger, David Coomes, Jonathan Lenoir, Leen Depauw, Sybryn L. Maes, Monika Wulf, Keith J. Kirby, Jörg Brunet, Martin Kopecký, František Máliš, Wolfgang Schmidt, Steffi Heinrichs, Jan den Ouden, Bogdan Jaroszewicz, Gauthier Buyse, Fabien Spicher, Kris Verheyen, Pieter De Frenne

**Affiliations:** ^1^ Forest Ecology and Conservation Group, Department of Plant Sciences University of Cambridge Cambridge UK; ^2^ Swiss Federal Institute for Forest, Snow and Landscape Research WSL Birmensdorf Switzerland; ^3^ UR “Ecologie et dynamique des systèmes anthropisés” (EDYSAN, UMR 7058 CNRS‐UPJV) Université de Picardie Jules Verne Amiens France; ^4^ Forest & Nature Lab, Department of Environment Ghent University Melle‐Gontrode Belgium; ^5^ Leibniz‐ZALF e.V. Müncheberg Müncheberg Germany; ^6^ Department of Plant Sciences University of Oxford Oxford UK; ^7^ Southern Swedish Forest Research Centre Swedish University of Agricultural Sciences Alnarp Sweden; ^8^ Institute of Botany Czech Academy of Sciences Průhonice Czech Republic; ^9^ Faculty of Forestry and Wood Sciences Czech University of Life Sciences Prague Czech Republic; ^10^ Faculty of Forestry Technical University in Zvolen Zvolen Slovakia; ^11^ Department Silviculture and Forest Ecology of the Temperate Zones University of Göttingen Göttingen Germany; ^12^ Forest Ecology and Forest Management Group Wageningen University Wageningen The Netherlands; ^13^ Białowieża Geobotanical Station Faculty of Biology University of Warsaw Białowieża Poland

**Keywords:** canopy density, climate change, forest composition, forest structure, global warming, macroclimate, microclimate, temperature buffering, understorey

## Abstract

**Aim:**

Forest understorey microclimates are often buffered against extreme heat or cold, with important implications for the organisms living in these environments. We quantified seasonal effects of understorey microclimate predictors describing canopy structure, canopy composition and topography (i.e., local factors) and the forest patch size and distance to the coast (i.e., landscape factors).

**Location:**

Temperate forests in Europe.

**Time period:**

2017–2018.

**Major taxa studied:**

Woody plants.

**Methods:**

We combined data from a microclimate sensor network with weather‐station records to calculate the difference, or offset, between temperatures measured inside and outside forests. We used regression analysis to study the effects of local and landscape factors on the seasonal offset of minimum, mean and maximum temperatures.

**Results:**

The maximum temperature during the summer was on average cooler by 2.1 °C inside than outside forests, and the minimum temperatures during the winter and spring were 0.4 and 0.9 °C warmer. The local canopy cover was a strong nonlinear driver of the maximum temperature offset during summer, and we found increased cooling beneath tree species that cast the deepest shade. Seasonal offsets of minimum temperature were mainly regulated by landscape and topographic features, such as the distance to the coast and topographic position.

**Main conclusions:**

Forest organisms experience less severe temperature extremes than suggested by currently available macroclimate data; therefore, climate–species relationships and the responses of species to anthropogenic global warming cannot be modelled accurately in forests using macroclimate data alone. Changes in canopy cover and composition will strongly modulate the warming of maximum temperatures in forest understories, with important implications for understanding the responses of forest biodiversity and functioning to the combined threats of land‐use change and climate change. Our predictive models are generally applicable across lowland temperate deciduous forests, providing ecologically important microclimate data for forest understories.

## 1 INTRODUCTION

The global network of standardized weather stations deliberately excludes forest microclimate, focusing instead on measuring synoptic, free‐air conditions that represent the macroclimate (De Frenne & Verheyen, 2016). Such weather stations are dictating the global climate data layers available for ecological research [e.g., CHELSA (Karger et al., 2017) and WorldClim (Fick & Hijmans, 2017)], despite the fact that such data do not represent well the climatic conditions that many forest organisms experience (Bramer et al., 2018; Potter, Woods, & Pincebourde, 2013). We thus know relatively little about forest microclimate gradients across large spatial scales and over time. This is a major impediment for global change biology, because forests cover almost one‐third of the land surface on Earth and harbour about two‐thirds of all terrestrial biodiversity (FAO, 2010; MEA, 2005).

Variation in forest structure, composition and topographic position leads to highly heterogeneous microclimate across space and time, with important consequences for the growth, survival and reproductive success of forest organisms and for forest functioning (Bazzaz & Wayne, 1994). The significance of microclimate has been acknowledged by ecologists and foresters for a long time, and microclimate is increasingly recognized as an important moderator of biotic responses to anthropogenic climate change (Geiger, Aron, & Todhunter, 2003; Lenoir, Hattab, & Pierre, 2017; Uvarov, 1931). For example, canopy structure and the associated microclimatic conditions strongly mediate the responses of forest species to climate warming (De Frenne et al., 2013; Scheffers, Edwards, Diesmos, Williams, & Evans, 2014). Locally experienced warming rates attributable to anthropogenic climate and land‐use change are strongly modified by changes in canopy structure (e.g., by changes in canopy cover). Quantification of the variability of forest temperature in space and over time will thus be key to addressing the responses of forest organisms to climate and land‐use change (Lenoir et al., 2017).

One potential route to derive forest microclimate dynamics is to infer them from climate data available from weather stations. Advanced modelling approaches, such as the mechanistic downscaling of microclimate from interpolated weather‐station data, make it increasingly feasible to approximate microclimate across space and over time (Bramer et al., 2018; Zellweger, Frenne, Lenoir, Rocchini, & Coomes, 2019). However, attempts to model forest microclimates are rare and often lack appropriate data for model calibration and validation (Kearney & Porter, 2017; Maclean et al., 2019). We need empirical, generalizable data at large spatial scales to further our understanding of the drivers of the differences between climatic measurements made inside forests and those made by nearby weather stations outside forests (Jucker et al., 2018). These could then be combined with the wealth of data describing forest structure and composition (e.g., collected within national forest inventories) to pave the way to translating past, present and projected macroclimate data into better representations of the climate conditions that forest organisms experience (Bramer et al., 2018). Nonetheless, quantitative assessments of forest microclimates at broad spatial scales and over sufficient timespans to detect seasonal effect sizes of key drivers of microclimate are still scarce (Greiser, Meineri, Luoto, Ehrlén, & Hylander, 2018).

Across all major biomes, understorey temperatures are offset to free‐air conditions by 1–4 °C or more, resulting in buffered (i.e., less extreme) temperature regimes below tree canopies (De Frenne et al., 2019). Maximum daytime temperatures in woodland understories are cooled by tree canopies, because they reduce transmission of short‐wave solar radiation to the understorey and cool the air by transpiration (Davis, Dobrowski, Holden, Higuera, & Abatzoglou, 2019). Tree canopies reduce radiative heat loss and emit some of the energy absorbed during the day to the understorey, thereby causing warmer daily minimum temperatures in the understorey compared with free‐air conditions (Geiger et al., 2003). Although less often studied, canopy composition may also affect the microclimate, because the quality and quantity of light transmitted by canopy foliage varies among tree species, leading to subtle species‐specific effects on the light conditions and associated microclimates (Renaud & Rebetez, 2009). However, despite a growing number of studies showing that canopy cover, basal area and/or canopy height are major determinants of understorey temperatures (Chen et al., 1999; Greiser et al., 2018; Jucker et al., 2018; von Arx, Graf Pannatier, Thimonier, Rebetez, & Gilliam, 2013), we still lack a general model of the form of the relationship at continental scales.

Differences between macro‐ and microclimate (i.e., temperature offsets) result from processes operating at many scales, and their influence may change over the course of the seasons. Topographic position and slope exposure have strong influences on radiation regimes and microclimatic gradients; for example, cold air drainage lowers daily minimum temperatures in areas where cold air flows and settles (Daly, Conklin, & Unsworth, 2010), resulting in increased temperature offsets (Lenoir et al., 2017). Such effects represent the influence of regional terrain features on local climate dynamics and are expected to be largely independent from effects brought about by local canopy characteristics. Wind mixes the air and reduces the differences between the macro‐ and microclimate. The levels of air mixing and lateral transfer of humidity and heat by wind generally decrease with increasing distance from the coast, from the edge of forest patches, or with increasing forest structural complexity, leading to increased temperature offsets (Bramer et al., 2018; Kovács, Tinya, & Ódor, 2017). At continental and global scales, the magnitude of the temperature offset varies considerably across biomes and forest types, suggesting that the macroclimate might explain some of the variation in microclimatic buffering (De Frenne et al., 2019). To put the influence of local drivers of microclimate into perspective, it will thus be important to study potential drivers at multiple spatial and temporal scales and to make systematic measurements at continental scales.

Here, we quantify the differences between air temperatures measured in the understorey and nearby weather stations in sites spanning much of the temperate deciduous forest biome of Europe. We analyse the seasonal variation in these temperature differences and compare the relative importance of local canopy structure and composition versus variables describing the landscape structure and the topography to explain this variability.

## 2 MATERIALS AND METHODS

### 2.1 Sampling design and study sites

**Figure 1:**
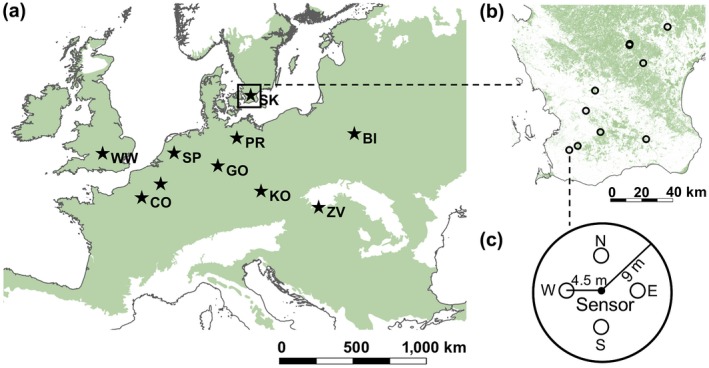
Sampling design, showing: (a) the distribution of the 10 sampled regions across the temperate deciduous forest biome in Europe (green area); (b) an example region (SK) and its forest cover taken from Hansen et al. (2013), with 10 plots spread along the regional gradient of canopy cover; (c) the plot sampling design, with the four interpretation points in each cardinal direction, as described in the main text. BI, Bialowieza; CO, Compiègne; GO, Göttingen; KO, Koda; PR, Prignitz; SK, Skane; SP, Speulderbos; TB, Tournibus; WW, Wytham; ZV, Zvolen [Colour figure can be viewed at https://www.wileyonlinelibrary.com]

We compiled data from 10 regions spanning an east–west gradient of *c*. 1,700 km and a north–south gradient of *c*. 800 km across a major part of the European temperate deciduous forest biome (Figure 1). In each region, we selected 10 plots representing a regional gradient of canopy cover. This resulted in 100 plots varying in total canopy cover (cumulative sum across all species and vertical layers) from as little as 41% up to 213%. The dominant tree species in terms of cover (with the number of plots in which they occur) were *Fagus sylvatica* (47), *Carpinus betulus* (44), *Fraxinus excelsior* (39), *Quercus robur* (34) and* Quercus petraea* (30). The mean annual temperature and precipitation during the time period 1979–2013 ranged from 7.3 to 11.0 °C and from 468 to 1,000 mm, respectively, across the studied regions (Karger et al., 2017).

### 2.2 Measurement of temperature and dependent variables

In each plot, we recorded air temperature every hour from 22 February 2017 to 21 February 2018, using Lascar Easy Log EL‐USB‐1 temperature sensors with an accuracy of ± 0.5 °C. The sensors were attached to a tree trunk with diameter at breast height (d.b.h.) > 25 cm at 1 m above the ground, which marked the centre of the plot (Figure 1c). To exclude potential bias attributable to direct sunlight, we placed the loggers in 18‐cm‐long white plastic radiation shields, which we attached at the north side of the tree trunk (see Supporting Information Figure S1, in Appendix S1). We aggregated the hourly temperature data to three daily temperature statistics: minimum daily (*T*_min_), mean daily (*T*_mean_) and maximum (*T*_max_) daily temperature. All daily time series were plotted, visually checked for obvious outliers and compared with all other times series within the respective region, including the respective temperature time series that we obtained from the closest weather station. This allowed us to verify and exclude sampling periods that were potentially biased owing to temporary device malfunction or misplacement (e.g., logger found on the ground owing to disturbance from wild boar, bear, deer, etc.). As a result, our sample sizes for spring, summer, autumn and winter were 92, 96, 95 and 98 plots, respectively.

We defined temperature offset values as the difference between the daily temperature statistics (*T*_min_, *T*_mean_ and *T*_max_) recorded inside the forest and the respective temperature statistic recorded by the closest official weather station representing free‐air conditions outside forests. The temperature offsets for *T*_min_, *T*_mean_ and *T*_max_ are our dependent variables. Negative offsets thus indicate cooler temperatures and positive offset values warmer temperatures inside versus outside forests. We focus on temperature offsets rather than absolute values to facilitate among‐region comparisons across Europe, because temperature differences between the macroclimate and the microclimate are most relevant for the responses of species to climate change, and because temporal temperature changes owing to anthropogenic climate change are also expressed against a baseline.

To account for temperature differences attributable to differences in elevation between the locations of the sensor and the weather station, we applied a constant lapse rate of 0.5 °C per 100 m for *T*_min_ and *T*_mean_, and a seasonal lapse rate for *T*_max_ of 0.5 °C in winter, 0.7 °C in spring and summer, and 0.6 °C in autumn. The choice of lapse rates was guided by empirical evidence from several regions in Europe (Kollas, Randin, Vitasse, & Körner, 2014; Rolland, 2003). Our study focus lies on lowland forests, and the differences in elevation between the plots and weather stations ranged between 1 and 284 m, with a median of 35 m (Supporting Information Appendix S2). Although lapse rates may vary between sites, seasons and temperature statistics (*T*_min_, *T*_mean_ and *T*_max_), such unaccounted variation in lapse rates would result in only minor differences in offset values, not affecting our main findings and conclusions. This is supported empirically by a lack of residual correlation of our models and data with the elevational differences between locations of the sensor and the weather station (Supporting Information Appendix S2).

We aggregated daily temperature offsets to calculate monthly means, in addition to means across the meteorological seasons [i.e., spring (March, April and May), summer (June, July and August), autumn (September, October and November) and winter (December, January and February)]. Absolute minimum temperatures can be a crucial factor limiting plant survival; therefore, we calculated the offset value for the absolute daily minimum temperature during winter, in addition to during spring (Kollas, Körner, & Randin, 2013).

### 2.3 Measurement of explanatory variables

We applied a combination of field‐based surveys and published spatial data to derive two groups of explanatory variables representing (a) local canopy structure and composition versus (b) landscape structure and topography (Table 1). Local‐scale canopy structure and composition was assessed between 3 July and 15 August 2017, within a circular plot area with a radius of 9 m around the central tree on which the temperature sensor was attached (Figure 1c). The plot dimensions were measured with a vertex hypsometer (Vertex IV), and the location of the interpretation point in each cardinal direction was marked with a pole. The coordinates of the plot centre were recorded using a differential global positioning system with an accuracy of *c.* 1 m. In each cardinal direction, we estimated canopy cover visually, by adding up the species‐specific vertical covers of all the plant species in the shrub and tree layer. The shrub and tree layers included all trees and shrubs with heights between 1 and 7 m, and > 7 m, respectively.

**Table 1:** Overview and summary statistics of predictor variables used to explain understorey temperature offsets

Variable group	Variable name	Description	Range (mean)	Unit
Local canopy structure and composition				
	Canopy cover	Visual estimation of vertical cover of shrub and tree layers, summed per species	41–213 (112)	Percentage
	Canopy openness	Total number of quadrats of open sky visible on spherical densiometer	3.9–59.50 (15.7)	Number
	Basal area	Basal area of trees with d.b.h. > 7.5 cm	5.2–122.3 (33.2)	Square metres per hectare
	Crown area	Predicted crown area per plot based on scaling relationships with d.b.h. (Jucker et al., 2016)	53.4–1,199 (309.1)	Square metres
	Tree height	Height of tree on which temperature sensor was placed; measured using a vertex hypsometer (Vertex IV)	9.2–40.0 (26.2)	Metres
	Shade‐casting ability	Tree species‐specific shade‐casting ability based on (Verheyen et al., 2012), community‐level mean index weighted by tree species‐specific canopy cover	2.1–5 (3.6)	From one (tree species with very open canopy) to five (very dense and shady species)
Landscape structure and topography				
	Forest cover	Proportion of area covered by forest within a circular buffer area with a radius of 250 m (Hansen et al., 2013)	18.1–100.0 (96.3)	Percentage
	Distance to forest edge	Distance to nearest forest edge (Hansen et al., 2013)	1.0–728.3 (119)	Metres
	Northness	Cosine of topographic aspect. Northness is a continuous variable describing the topographic exposition ranging from completely north exposed (−1) to completely south exposed (1)	−1.0 to 1.0 (−.3)	Index
	Slope	Topographic slope	0.4–22.0 (4.3)	Degrees
	Elevation	Elevation above sea level	30.7–636.9 (165.7)	Metres
	Topographic position	Relative topographic position describing the plot elevation in relationship to the surrounding elevations. Valley bottoms have low values; elevated locations, such as ridges, have high values	1.6–147.3 (23.5)	Metres
	Distance to coast	Distance to nearest coastline derived from Natural Earth (free vector and raster map data from naturalearthdata.com)	11.6–518.7 (107.6)	Kilometres

Note

Northness, slope, elevation and topographic position were derived from EU‐DEM (2018). Note that high values of basal area and crown area derive from inclusion of some large trees at the edge of the plots. d.b.h. = diameter at breast height.

The canopy cover per plot was then calculated as the mean of these four estimations. The species‐level approach for estimating canopy cover provides a detailed measure of the cumulative sum of cover across all species and vertical layers, allowing values to exceed 100% owing to overlaps. At the stand level, however, canopy cover estimates are often confined within the range of 0–100%. We therefore also analysed a transformed version of our canopy cover values by accounting for the overlap and constraining the cumulative cover values below 100% (for details, see Supporting Information Appendix S3).

Canopy openness was measured by taking the mean of spherical densiometer readings taken in the four sub‐plots. We used a concave spherical densiometer, which displays large parts of the sky hemisphere, thus enabling us to take an angular view for estimating the fraction of sky hemisphere not covered by the canopy (Baudry, Charmetant, Collet, & Ponette, 2014). It is important to note that our estimates of canopy cover and canopy openness represent one snapshot in time, neglecting temporal variation in leaf area and associated effects on microclimates.

Basal area was estimated based on the d.b.h. of all trees within the plot with a minimal d.b.h. of 7.5 cm, as measured with callipers.

The total sum of projected crown area (CA) for all individual tree species was estimated based on the allometric relationship between CA and d.b.h. (Jucker et al., 2016; for details, see Supporting Information Appendix S4). We considered CA as an additional variable because its link to microclimate is more mechanistic compared to d.b.h.

The height of the tree on which the temperature logger was attached was measured by the mean of two measurements from opposing directions using the vertex hypsometer (Vertex IV).

The shade‐casting ability (SCA) describes the ability of each tree species to cast a specific level of shade, ranging between one (very low SCA, e.g., *Betula* spp.) and five (very high SCA, e.g., *Fagus sylvatica*) (Verheyen et al., 2012). We calculated a weighted SCA per plot by using the species‐specific canopy cover estimates as weights. This allowed us to test whether canopies composed of tree species with higher SCA scores have a stronger offsetting capacity than those with low SCA scores.

Landscape and topographic characteristics were derived from satellite‐based global tree cover data with a spatial resolution of *c*. 30 m (Hansen et al., 2013) and a pan‐European digital elevation model (DEM) with a spatial resolution of 25 m, using Copernicus data and information from the European Union (EU‐DEM, 2018).

Forest cover was assessed within a circular buffer area with a radius of 250 m and measured as the percentage of area covered by a minimum tree cover of 20% (Hansen et al., 2013).

Distance to the forest edge was calculated by transforming the forest cover mask into contour lines and extracting the distance from the plot coordinate to the nearest contour line, using the *raster To Contour* and *g Distance *functions in the R packages “raster” (Hijmans, 2017) and “rgeos” (Bivand & Rundel, 2018). Landscape‐level forest cover and distance to edge have previously been related to forest microclimates (Greiser et al., 2018; Latimer & Zuckerberg, 2017) and may affect the level of air mixing and the lateral transfer of heat and humidity by wind, thus affecting the temperature offset.

Topographic northness, slope, elevation and topographic position were all derived from the DEM to represent topographic effects on the offset of understorey temperatures, including variation in solar radiation incidence and cold air drainage, an important process affecting minimum temperatures at night and during the winter (Ashcroft & Gollan, 2013; Daly et al., 2010). Topographic northness describes the topographic exposition, ranging from completely north exposed to completely south exposed, and was derived as the cosine of topographic aspect. Topographic position was calculated as the difference between the elevation of the plot cell and the lowest cell within a circular buffer area with a radius of 500 m (Ashcroft & Gollan, 2013).

We also considered the distance to the nearest coastline, because the temperature offset may increase with increasing distance to the coast, owing to increased temperature ranges and lower levels of air mixing.

### 2.4 Statistical analysis

To analyse the relative importance of our two groups of predictor variables (i.e., local canopy characteristics versus landscape‐level metrics) for explaining temperature offsets, we used variation partitioning, following Borcard, Legendre and Drapeau (1992). First, we performed a principal components analysis (PCA) for each of the variable groups and used the first two axes per group as predictor variables in the subsequent analysis. Thus, the number of predictor variables used per group was the same. Among canopy characteristics, crown area and canopy cover had the highest loadings on the first and second PCA axis, respectively, whereas the loadings for the landscape metrics were more variable among predictor variables (Supporting Information Appendix S5). We then fitted linear mixed‐effects models (LMMs) with the PCA axes as fixed effects and “region” as a random intercept term to account for the non‐independence among replicates from the same region, using restricted maximum likelihood in the *lmer* function from the lme4 package (Bates et al., 2015). We did not include a random slope term because it resulted in higher Akaike information criterion values when compared with the models with random intercepts only. We fitted three LMMs: one for each of the two variable groups (local canopy characteristics versus landscape‐level metrics) and one for the combination of both groups. Based on these three LMMs, we finally partitioned the amount of explained variation (marginal *R* ^2^) into individual and shared fractions (Borcard et al., 1992).

To report the relationship between each individual predictor variable and each dependent variable (i.e., the offset values for *T*_min_, *T*_mean_ and *T*_max_), we performed χ^2^ tests by comparing the univariate LMM including each single predictor (scaled to a mean of zero and *SD* of one) with a respective intercept‐only model, both with “region” as a random intercept term (Zuur, Ieno, Walker, Saveliev, & Smith, 2009). We ln‐transformed canopy openness and topographic position to conform better to normality. Goodness‐of‐fit was determined by calculating marginal and conditional *R* ^2^ values (following Nakagawa & Schielzeth, 2012) using the *r.squaredGLMM* function in the MuMIn‐package (Barton, 2018). The marginal *R* ^2 ^describes the variation explained by the fixed factors only, whereas the conditional *R* ^2^ describes the variation explained by the fixed and random factors together (Nakagawa & Schielzeth, 2012). [Correction statement added on 23 Oct 2019 after first online publication: “Log_10_‐transformed” was changed to “ln‐transformed” in this paragraph]

We expected that the random intercept term “region” would capture major gradients in macroclimate in our sampling design (Figure 1), leaving little variation in temperature offset to be explained by macroclimate once regional effects had been accounted for. To test this assumption, we performed an additional variation partitioning exercise with three variables groups (i.e., the two groups representing local canopy characteristics and landscape‐level metrics and an additional group representing the macroclimate). The variables in the latter group were the long‐term (1979–2013) mean annual precipitation and temperature (Karger et al., 2017), in addition to the daily minimum, maximum and mean temperature statistics from the weather stations for the 1‐year period matching with the understorey data from the temperature sensors, aggregated over the same time periods as the dependent variables. Following the approach chosen for the two other groups of local canopy characteristics and landscape‐level metrics and to ensure that the number of predictor variables used per group was the same, we used the first two axes of a PCA on macroclimate variables as predictor variables in the variation partitioning (Supporting Information Appendix S5).

To test for nonlinear relationships between the temperature offset and canopy characteristics, in addition to topographic position, we used general additive mixed‐effects models (GAMMs) with the *gamm *function in the “mgcv” package (Wood, 2017), and again “region” was added as random term. To complement the nonlinearity check and to identify possible break points or thresholds, we used piecewise regression based on the function *segmented* in the “segmented” package (Muggeo, 2017).

To investigate the degree to which the relationships between canopy characteristics and temperature offset are transferable to other regions across the temperate deciduous forest biome, we assessed the predictive performance of the model based on a cross‐validation procedure with blocked data splitting, accounting for our hierarchical sampling design (“region” as a random effect) (Roberts et al., 2017). To this end, we calibrated 10 different models for each of the six canopy variables (i.e., 60 models in total). Each model was calibrated using the data from nine regions and validated based on the predictions made to the 10th, left‐out region. For the sake of parsimony, we combined each canopy variable with only one variable describing landscape structure and topography (i.e., distance to the coast), which had a relatively large influence on the magnitude of the offset value for maximum temperatures (see Results). We refrained from analysing the predictive performance of the landscape structure and topography variables, because our focus here was primarily on the effects of the canopy structure and composition. Canopy variables were relatively unimportant for explaining variation in the offset of *T*_min_; therefore, we restricted our analysis to *T*_max_. Predictive performance was assessed based on the *R* ^2^ value comparing the predicted versus the observed values. All analyses were performed in R v.3.5.0 (R Core Team, 2018).

## 3 RESULTS

**Figure 2:**
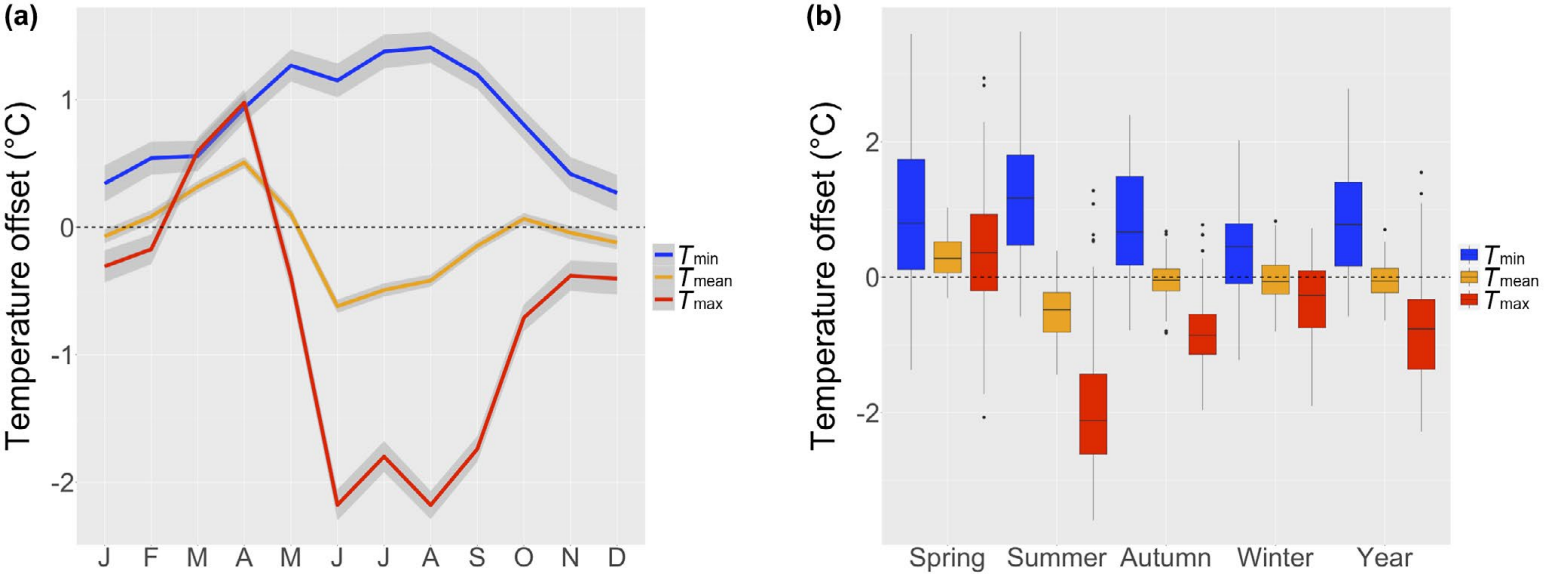
(a) Daily air temperature offsets per month with 95% confidence intervals (grey ribbons), measured during 1 year in the understorey of temperate deciduous forests in Europe (Figure 1). (b) Distributions of temperature offset values during spring (March–May), summer (June–August), autumn (September–November), winter (December–February) and the entire year. Positive values indicate warmer conditions and negative values cooler conditions in the understorey compared with nearby free‐air conditions measured by weather stations [Colour figure can be viewed at https://www.wileyonlinelibrary.com]

The mean (range) daily maximum air temperature (*T*_max_) offset during summer was −2.1 °C (−3.7 to 1.4) and mean daily minimum air temperature (*T*_min_) offset during winter was 0.4 °C (−1.2 to 2.0) (Figure 2). Across all regions and the whole year, the mean offset of *T*_max_ and *T*_min_ was −0.8 °C (−2.3 to 1.6) and 0.9 °C (−0.6 to 2.8), respectively. The offset of daily average temperatures (*T*_mean_) was generally low, with means of −0.5 °C (−1.4 to 0.4) during summer and −0.03 °C (−0.8 to 0.8) during winter.

The offset of temperature extremes varied considerably between the sampled regions and months and seasons, and was most pronounced during summer and least distinctive during winter (Figure 2; Supporting Information Appendix S6). Interestingly, the offset of *T*_max_ during spring was slightly positive, with a mean of 0.4 °C (−2.4 to 3.0), indicating that spring *T*_max_ inside forests may often be higher, not lower, than outside forests. The average offset of *T*_min_ in spring was also positive [i.e., mean daily minimum temperatures in spring were warmer by 0.9 °C (−1.4 to 3.6) in the understorey than outside forests]. The same pattern was found for absolute daily minimum temperature offset during spring and winter, with means of 0.9 °C (−1.7 to 3.2) and 1.5 °C (−1.1 to 5.4), respectively (Supporting Information Appendix S7).

**Figure 3:**
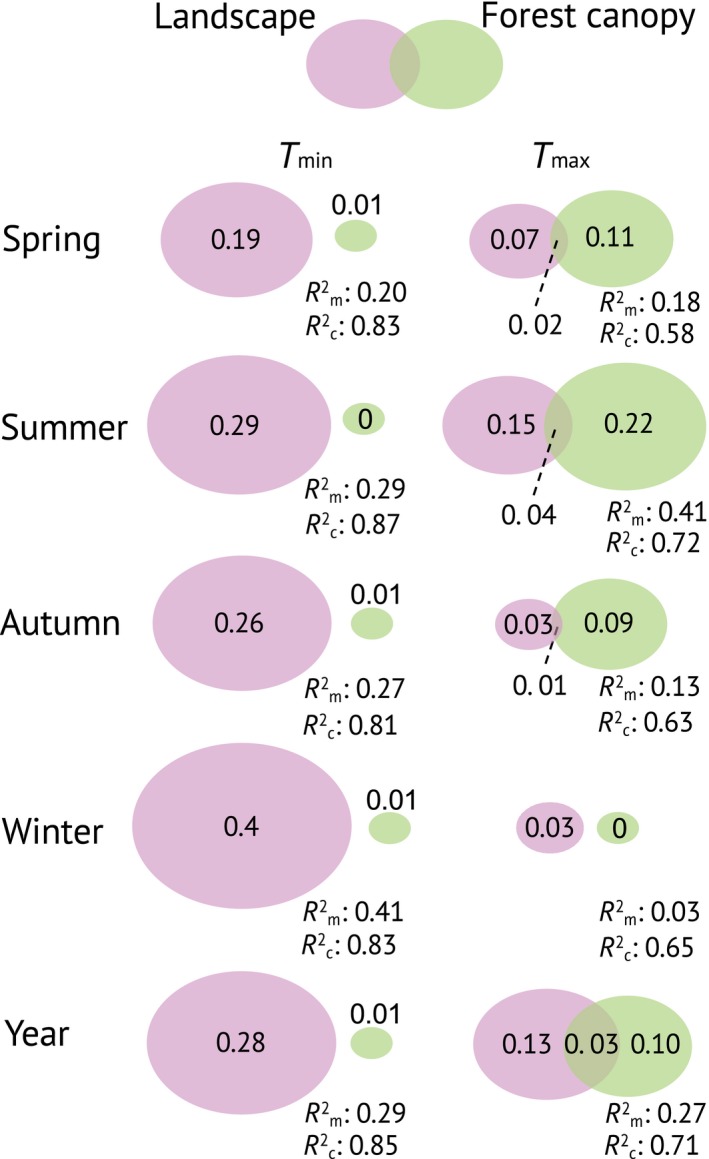
Venn–Euler diagrams showing the independent share of explained variation [marginal *R* ^2^ (*R* ^2^_m_)] for each variable group (i.e., landscape and forest canopy), in addition to the shared amount of explained variation (intersection of ellipses), as determined by variation partitioning. The sizes of the ellipses are scaled according to *R* ^2^_m_. The *R* ^2^_m_ describes the variation explained by fixed factors only, whereas conditional *R* ^2^ (*R* ^2^_c_) is the variation explained by the fixed and random factors together [Colour figure can be viewed at https://www.wileyonlinelibrary.com]

Partitioning the explained variance into independent contributions of local canopy characteristics versus landscape and topography metrics, in addition to their joint contributions, showed that canopy characteristics were generally more important for explaining the variation in *T*_max_ offsets, whereas landscape and topography metrics were most important for explaining *T*_min_ offsets (Figure 3). During summer, the independent effect of canopy characteristics on *T*_max_ offset was greatest, with a marginal *R* ^2^ = .22. During winter, landscape and topography metrics independently explained 40% of the variation (marginal *R* ^2^ = .4) in *T*_min_ offset. The joint contributions between canopy characteristics and landscape and topography metrics were low, suggesting that the groups capture different processes governing forest microclimates. The total marginal *R* ^2^ values for *T*_max_ offset during summer and *T*_min_ offset during winter were both .41, and thus considerably higher than the *R* ^2^ values for *T*_min_ and *T*_max_ offset during spring and autumn, which ranged between .13 and .27 (Figure 3). In line with our expectation, including the macroclimate as a third variable group in the variation partitioning revealed relatively small independent effects of macroclimate, except for *T*_min_ in spring (Supporting Information Appendix S8 and Figure S8).

**Figure 4:**
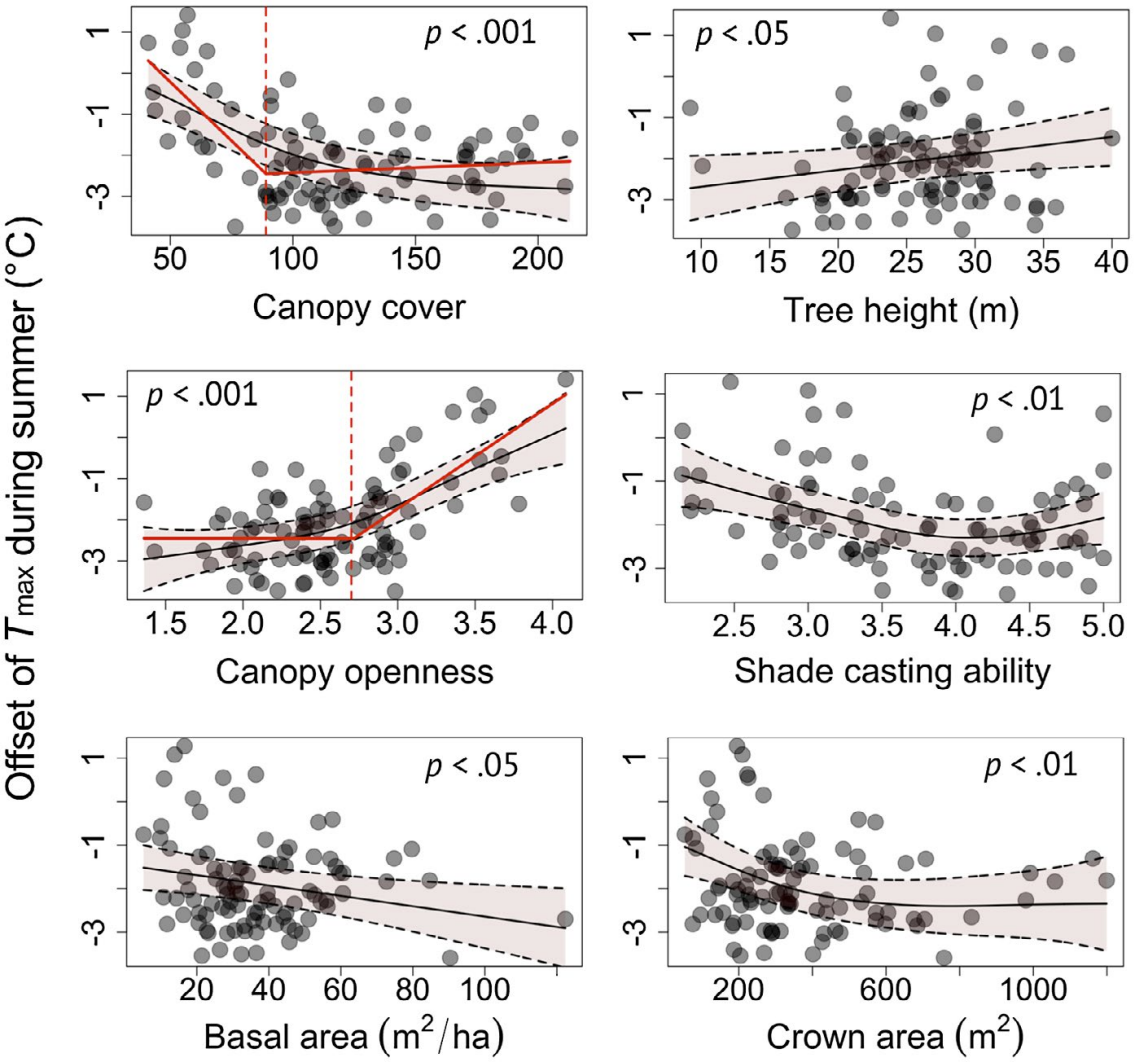
Relationships between canopy characteristics and the offset of daily maximum temperatures during summer. Smoothed curves with 95% confidence intervals (light red polygons) and *p*‐values from the general additive mixed‐effects models. Canopy openness was ln‐transformed. Canopy cover and canopy openness show nonlinear relationships, with break points at 89% and 2.7, respectively, as indicated by the dashed lines. The continuous red lines show the regression lines as calculated using piecewise regression (see main text for details). We did not elaborate on threshold effects for shade‐casting ability and crown area because of the large confidence intervals. Positive offset values represent warmer temperatures inside than outside forests; negative offset values indicate cooler temperatures inside than outside forests [Correction statement added on 23 Oct 2019 after first online publication: “Log_10_‐transformed” was changed to “ln‐transformed” in the caption for Figure 4] [Colour figure can be viewed at https://www.wileyonlinelibrary.com]

Analysis of the independent effect of canopy characteristics on the offset of *T*_max_ during summer revealed a negative and nonlinear relationship for canopy cover (i.e., the cooling of *T*_max_ in the understorey increased nonlinearly with increasing canopy cover; Figure 4). Piecewise regression analysis identified a canopy cover threshold at 89% (*SE* 8.5%), below which the offsetting capacity of canopy cover increased rapidly when additional vegetation cover was added. The results for the transformed version of canopy cover with values constrained to range between 0 and 100% suggest a threshold of 75% (*SE* 5.2%) and a comparably weak nonlinearity (Supporting Information Appendix S3). Nonlinear relationships were also found for canopy openness and crown area, but not for basal area, which was related weakly and negatively to the offset of *T*_max_ during summer (Figure 4; Supporting Information Appendix S9 Table S9). Contrary to our expectations, the *T*_max_ offset increased with increasing tree height, suggesting a decrease in temperature buffering. However, this relationship was weak, and we therefore refrain from further interpretation of this result.

The SCA of the tree species composition was related significantly and negatively to the offset of *T*_max_, indicating that the buffering capacity increases with increasing SCA (Figure 4). Shade‐casting ability was not correlated with any of the canopy structure metrics tested, suggesting that the canopy composition holds information for explaining the temperature offset that is complementary to canopy structure (Supporting Information Appendix S10).

**Figure 5:**
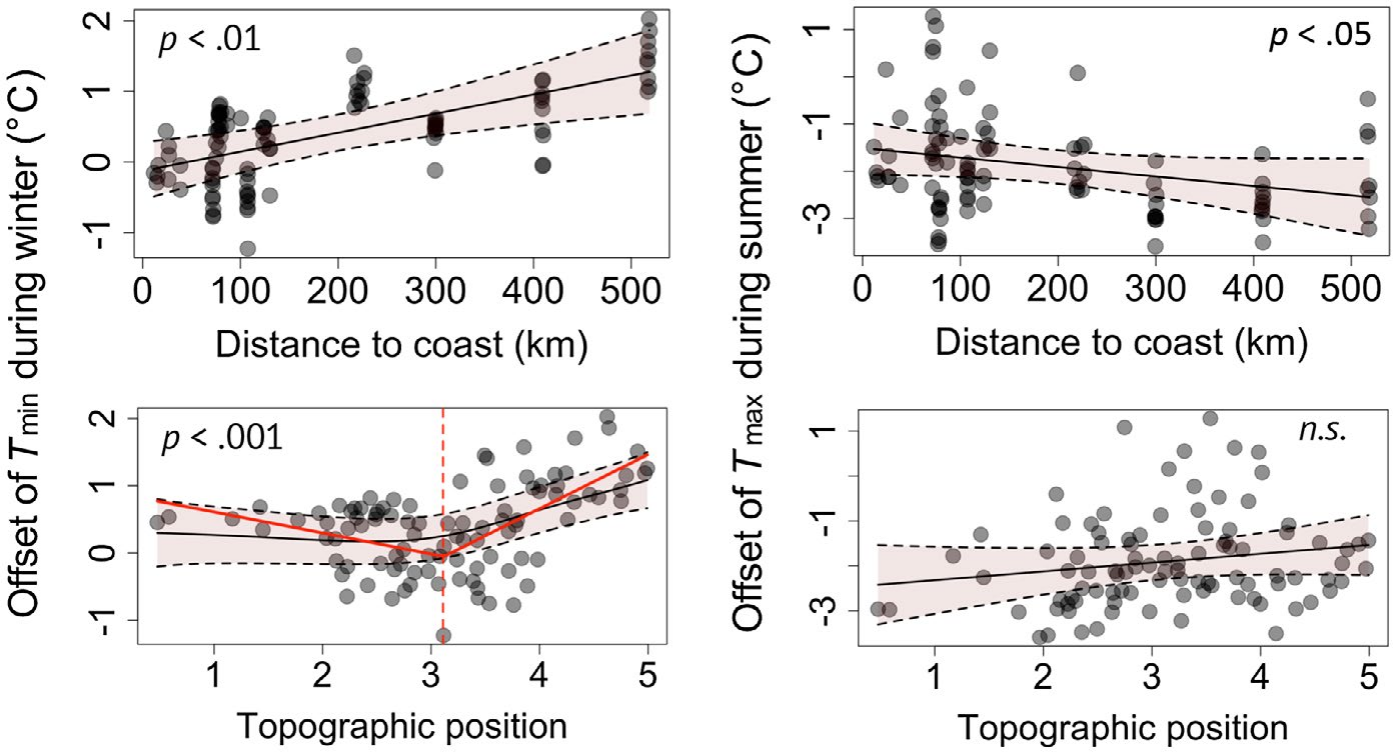
Relationships between the distance to the coast and relative topographic position (ln‐transformed, with low values representing valley bottoms and high values representing elevated locations, e.g., ridges) and the offset of daily minimum temperatures during winter, and daily maximum temperatures during summer. Topographic position was related nonlinearly to *T*_min_ offset during winter, with a threshold at 3.1 (*SE* 0.16), as indicated by the red dashed line. The 95% confidence intervals (light red polygons) and *p*‐values from the general additive mixed‐effects models are shown. Positive offset values represent warmer temperatures inside than outside forests; negative offset values indicate cooler temperatures inside than outside forests [Correction statement added on 23 Oct 2019 after first online publication: “n.s.” was changed to “*p* < .05” in the top right image of Figure 5 and “Log_10_‐transformed” was changed to “ln‐transformed” in the caption for Figure 5] [Colour figure can be viewed at https://www.wileyonlinelibrary.com]

The topographic position, distance to the coast and elevation were the most important predictors for *T*_min_ offset across the seasons (Supporting Information Table S8). The minimum temperature offsets increased linearly with increasing distance to the coast, explaining 39% of the variation for *T*_min_ during winter and 17% of the variation for *T*_max_ during summer (Figure 5; Supporting Information Table S8). Elevation and distance to the coast were strongly correlated (Pearson's *r*: .84; Supporting Information Appendix S10 and Figure S10) and thus showed similar patterns. We therefore do not elaborate further on the effects of elevation on the temperature offset. Topographic position was nonlinearly related to the offset of *T*_min_ in winter (Figure 5) and was also an important predictor of the offset of the absolute daily minimum temperature in winter and spring (Supporting Information Table S6). Landscape‐level forest cover and distance to the nearest forest edge were equally unimportant for explaining understorey temperature offsets (Supporting Information Table S8).

Cross‐validation of our models suggests that the GAMMs including canopy cover or canopy openness predict the offset of *T*_max_ during the summer reasonably well, with marginal *R* ^2^ values of .33 and .43, respectively (Supporting Information Appendix S11). These results further support the nonlinear relationship between canopy cover and *T*_max_ offset; the marginal *R* ^2^ value from the linear models (i.e., LMMs) including canopy cover was .24 and thus considerably lower than that of the GAMMs (.33). However, the opposite was the case for canopy openness, with *R* ^2^ values of .43 and .24, respectively. Shade‐casting ability also had a moderate predictive performance, with a marginal *R* ^2^ value from cross‐validated GAMMs of .20 for the offset of *T*_max_ during summer. The predictive performances of basal area, crown area and tree height were low, with *R* ^2^ values ranging from .06 to .10 (Supporting Information Table S11).

## 4 DISCUSSION

Understorey air temperature extremes in temperate lowland deciduous forests across Europe are considerably less severe than (or buffered from) those reported by weather stations outside forests, with mean (range) summer maximum and winter minimum temperature offset values of −2.1 (−3.7 to 1.4) and 0.4 °C (−1.2 to 2.0), respectively. Together with the spatial and temporal analysis of the drivers of the temperature offset, our results have important implications for improving the analysis of forest microclimates and their effects on forest biodiversity and functioning in the context of climate warming and land‐use change.

Canopy structure and composition play a key role in regulating the offset of maximum summer temperatures. Forests thus provide highly heterogeneous thermal environments, with maximum temperature conditions that are often much cooler than suggested by available climate layers (Jucker et al., 2018; Scheffers et al., 2017; Senior, Hill, Benedick, & Edwards, 2018). The maximum temperature offsets reported here compare well with the general patterns observed in temperate regions across the globe and might even increase if the forest temperatures were to be measured closer to the forest ground surface (De Frenne et al., 2019). Local maximum temperatures matter greatly for the response of organisms to climate warming, because the relative fitness of a species is related strongly to the species‐specific heat tolerance (Huey et al., 2012). Many species living below tree canopies may therefore find thermal refuges within their habitats, allowing them to evade short‐term temperature extremes (Scheffers et al., 2014). Topographic microclimate heterogeneity and the associated provision of microrefugia reduce the climate‐change‐related extinction risk of plants and insects (Suggitt et al., 2018), and our microclimate results suggest that this might also apply in forests; data on organismal responses are needed to explore this issue further. The future provision of thermal refuges will depend on the degree to which microclimates are decoupled from the macroclimate, potentially resulting in different warming rates under the canopy versus in the open (De Frenne et al., 2019).

Changes in canopy structure and composition may alter local minimum and maximum temperatures at magnitudes exceeding the rates of macroclimate warming in the decades to come (IPCC, 2013). Habitat modifications resulting from a decrease of canopy cover (e.g., tree harvest in production forests) thus strongly intensify the local impact of macroclimate warming (and, conversely, increasing cover mitigates the impact), which has significant implications for forest biodiversity dynamics and functioning. Habitat modifications in favour of warmer habitats matter for the re‐assembly of terrestrial communities, because the heat tolerance varies among species, putting species with low heat tolerances at higher risk of being filtered out (Nowakowski et al., 2018). Incorporating canopy density information and associated shade effects into biophysical models of body temperatures is thus key to improving the predictions of the vulnerability of animals to climate change (Algar, Morley, & Boyd, 2018). Increasing forest density, as has been observed in many temperate European forests as a consequence of changes in forest management over the past decades (e.g., Hedl, Kopecký, & Komarek 2010), might have compensated for, or even reversed, recent increases in maximum temperatures arising from anthropogenic global warming in some of these forests. Temperature buffering by trees also directly impacts human health and well‐being (e.g., in cities, where trees alleviate human exposure to heat; Armson, Stringer, & Ennos, 2012). Consideration of the interactions between regional macroclimate warming and the local spatial and temporal dynamics in microclimates is crucial for the accurate assessment of the responses of forest biodiversity, ecosystem functioning and service provisioning to rapid global change.

The regulating effect of canopy structure and composition on understorey microclimate has long been embraced by forest ecologists and managers. Nevertheless, our finding that understorey maximum temperatures are also regulated by differences in the composition of deciduous tree species, owing to species‐specific shade‐casting abilities, provides new insights into the drivers of understorey microclimates. We also show that the offset of maximum understorey air temperatures is nonlinearly related to canopy structure (e.g., to canopy cover, a proxy variable for the understorey light conditions). Understorey temperature offsets may thus be tied closely to the nonlinear light absorption along the vertical canopy profile, as proposed by the Beer–Lambert law (Monsi & Saeki, 1953). Together with findings from the tropics (Jucker et al., 2018) and the temperate forests in Australia (Ashcroft & Gollan, 2012), which also showed nonlinear effects of canopy cover on maximum temperatures, our results suggest that such nonlinear relationships might represent a general and globally relevant phenomenon, providing important insights into the mechanisms governing forest microclimate gradients.

Forest managers and ecologists frequently use canopy structure per se (e.g., quantified via variables such as canopy cover, basal area and leaf area index (LAI) as a proxy for understorey microclimatic (including light) conditions, which are key drivers of forest regeneration and species performance. Accounting for nonlinear relationships between canopy structure, light availability and extreme temperatures with associated threshold effects might help forest managers to promote tree regeneration by creating or maintaining suitable tree species‐specific microclimatic conditions, or mitigate microclimate extremes and related damage to crops produced in agroforestry schemes (Lin, 2007). In particular, we found that canopy cover increases daily absolute minimum temperatures during the spring, confirming evidence that the risks of spring frost damage on tree regeneration are reduced under canopy (Kollas et al., 2013). Interpreting seasonal effects of canopy cover on microclimates would be based optimally on data representing the seasonal variation in canopy cover, the lack of which is a limitation to many studies, including ours. Investigation of the effects of temporal canopy cover dynamics on microclimates thus provides an interesting avenue for further research. Moreover, higher spring mean and maximum temperatures in forests compared with free‐air conditions might be driven by increased absorption of solar radiation by dark stems (bark) and remaining leaf litter, resulting in accelerated snow melting and prolonged growing seasons (Wild et al., 2014). Last, but not least, better knowledge about the relationship between canopy structure and microclimate will help to improve the ecological insights gained from investigations of forest structure–biodiversity relationships (Zellweger, Roth, Bugmann, & Bollmann, 2017) and will prove useful in attempts to maximize stepping stones and microrefugia in human‐dominated forest landscapes (Hannah et al., 2014).

Understorey temperatures are regulated by complementing effects of local canopy attributes and by topographic and landscape features derived at regional and landscape scales. Increasing daily and seasonal temperature ranges with increasing distance to the coast (continentality) result in higher offset values (e.g., owing to an increase in clear‐sky days). The effects of microclimate buffering can thus be expected to be highest in dense forests in continental regions. Topographic position includes the effects of cold air drainage and pooling, which drive minimum temperatures during night and winter, particularly in calm, still conditions (Ashcroft & Gollan, 2012; Daly et al., 2010; Dobrowski, 2011). Elevated locations inside forests may thus experience relatively warm temperatures, leading to longer snow‐free periods and longer vegetation periods than suggested by macroclimate layers. Lower temperatures at topographic depressions enable persistent snow cover during winter, allowing winter‐adapted plants and animals to overwinter in warmer and more stable conditions beneath the snow (Pauli, Zuckerberg, Whiteman, & Porter, 2013).

Our approach and analysis enable the approximation of forest temperatures based on widely available weather‐station data with high temporal resolution. Although mechanistic downscaling of macroclimate data might achieve the same goal (Maclean et al., 2019), our models can be used efficiently to predict understorey temperatures from weather‐station data, based on readily available that data about canopy structure and composition, in addition to topography and landscape characteristics. For example, multi‐temporal canopy cover data collected within forest inventories can be used directly to make plot‐level predictions of how forest microclimates have changed over time and how this is related to the responses of forest biodiversity and functioning to climate and land‐use change. Likewise, future scenarios of dynamics in canopy cover and composition can be incorporated into more realistic predictions of future forest climatic conditions and their ecological implications. Together with upcoming microclimate mapping techniques, such as the interpolation of in situ forest microclimate measurements using LiDAR remote sensing‐based canopy cover maps (Zellweger et al., 2019), the presented approach will be useful to fill the current gap in forest microclimate data (De Frenne & Verheyen, 2016).

## BIOSKETCH

We are broadly interested in the responses of forest biodiversity and functioning to climate and land‐use change. We are particularly interested in the role of forest microclimate dynamics in driving these responses.

## Data Availability

Data will be uploaded to a Dryad repository (doi:https://doi.org/10.5061/dryad.cv1jg30).
