# Supplementary material for: Seasonal drivers of understorey temperature buffering in temperate deciduous forests across Europe
Source: Glob Ecol Biogeogr. 2019 Aug 22;28(12):1774–86. doi: 10.1111/geb.12991 (PMC6900070; doi:10.1111/geb.12991)
Supplement: Supplementary file 1 [file GEB-28-1774-s001.docx]

**Supporting information**

[Appendix S1 | Temperature logger installation in the forest understorey 2](#_Toc7084246)

[Appendix S2 | Lapse rate sensitivity analysis and weather station data 3](#_Toc7084247)

[Appendix S3 | Estimation of Crown Area 6](#_Toc7084248)

[Appendix S4 | Principle components analysis 7](#_Toc7084249)

[Appendix S5 | Monthly temperature offset values per region 9](#_Toc7084250)

[Appendix S6 | Offset values of absolute minimum temperatures in spring and winter 10](#_Toc7084251)

[Appendix S7 | Extended results from variation partitioning 12](#_Toc7084252)

[Appendix S8 | Individual variable effects on the temperature offset 14](#_Toc7084253)

[Appendix S9 | Extended Canopy Cover analysis 19](#_Toc7084254)

[Appendix S10 | Correlation matrix and histograms of predictor variables 20](#_Toc7084255)

[Appendix S11 | Predictive performance of canopy variables for Tmax offset 21](#_Toc7084256)

## Appendix S1 | Temperature logger installation in the forest understorey


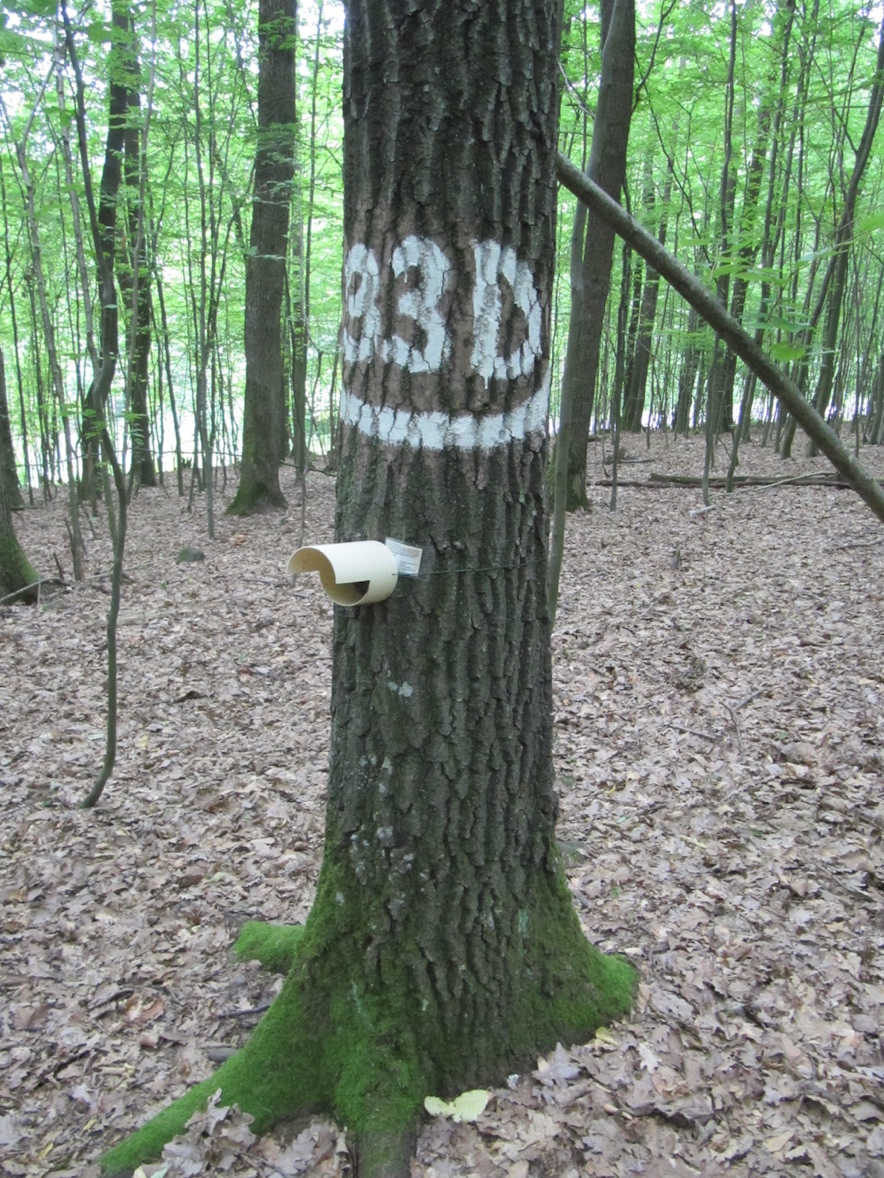


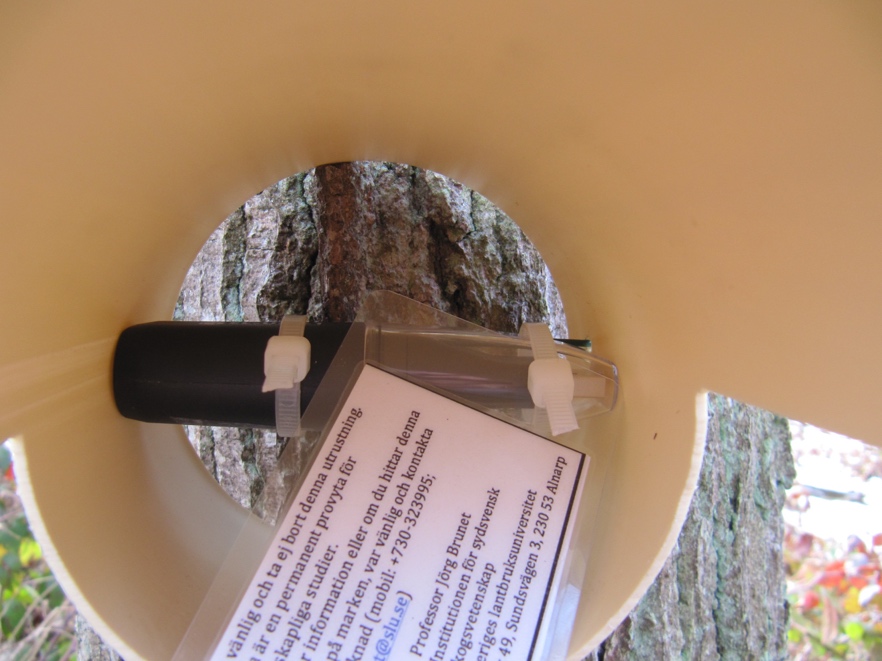


**Figure S1**. Temperature logger installation on the north side of a tree trunk at 1m height in a forest in Zvolen (ZV), Slovakia.

## Appendix S2 | Lapse rate sensitivity analysis and weather station data

The effects of using different lapse rate corrections were explored. To calculate the difference between the temperatures in the understorey and the free-air conditions measured outside forests, we downloaded the daily minimum, mean and maximum temperatures from the official weather station nearest to each plot.  The weather stations were located at different elevations to the plots, and given that temperature varies systematically with altitude due to atmospheric pressure changes (i.e. lapse rates), corrections were needed to make the datasets comparable. Lapse rates vary with season and geographic region (Rolland, 2003), and we explored different approaches.

As mentioned in the main manuscript, for Tmin and Tmean we applied a lapse rate of 0.5 °C to correct for temperature differences due to differences in elevation between the locations of the plots (sensor) and weather stations. For Tmax during spring, summer, autumn and winter we applied lapse rates of 0.7, 0.7, 0.6 and 0.5 °C, respectively. The focus of our paper lies on minimum and maximum temperatures, for which our lapse rates represent realistic assumptions guided by empirical evidence from different regions in Europe (Rolland, 2003; Kollas *et al.*, 2014).

The Tmax offset that we calculated by subtracting plot measurements from lapse-rate-adjusted weather station measurements is not correlated with the absolute difference in elevation, nor with the distance to the weather stations (Figure S2a), and there is no residual correlation with the difference in elevation (Figure S2b). This indicates that the difference in elevation does not bias our models for Tmax offset. The relationship between Tmin offset and the absolute difference in elevation followed a regional pattern, which we accounted for in our mixed effects modelling framework. Tmin offset is not correlated with distance to weather station (Figure S2a), and again there is no residual correlation with the difference in elevation (Figure S2b). The main conclusions drawn from the relationships between canopy cover and Tmax offsets are not affected by the different lapse rates, even when considering the dry adiabatic lapse rate of 1 °C per 100m (Figure S2c). We also recalculated all Tmax buffering values by replacing the weather station temperature data with interpolated climate data, i.e. TerraClimate (Abatzoglou *et al.*, 2018), and arrive at the same conclusions. The relationship between topographic position and the Tmin offset during winter stayed the same for lapse rates of 0.4 °C and 0.5 °C per 100 m, without any residual correlation with elevational differences (Figure S2d). We are therefore confident that our main findings and conclusions are not affected by unaccounted variation in different lapse rates.

One reason why are results are robust is that all plots and weather stations were located in the lowlands. The absolute difference in elevation between plots and weather stations ranged from 1.7 to 284 m, with a median of 35 m and mean of 69 m. The spatial distance between the weather stations and the plots ranged from 0.9 to 37.7 km, with a median of 12.4 and mean of 15.0 km.


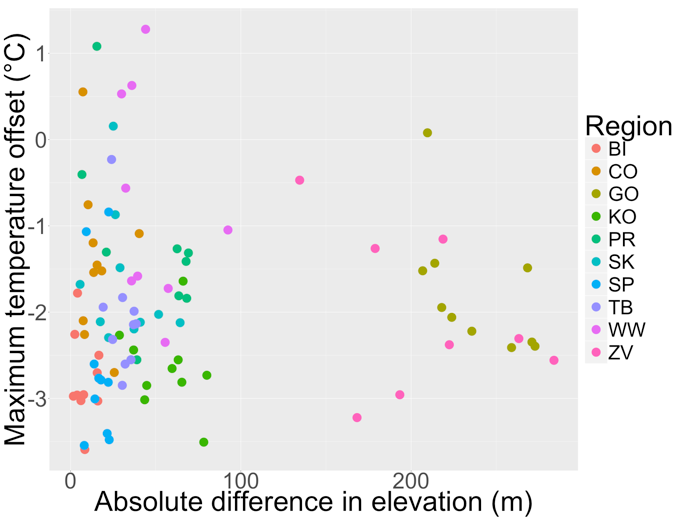

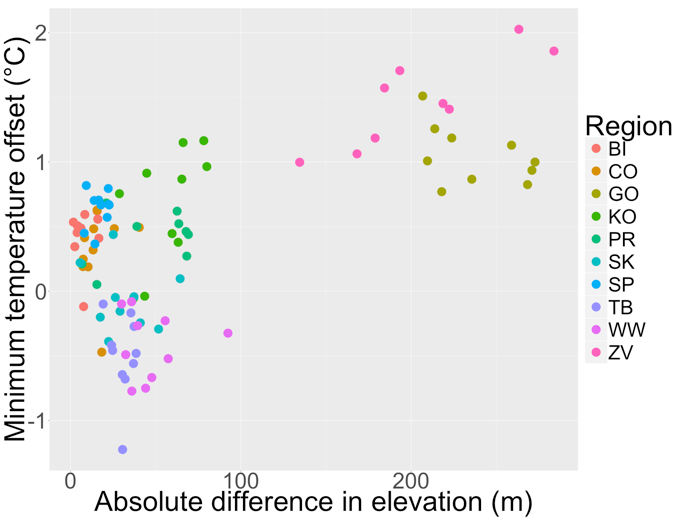


B

A

D

C
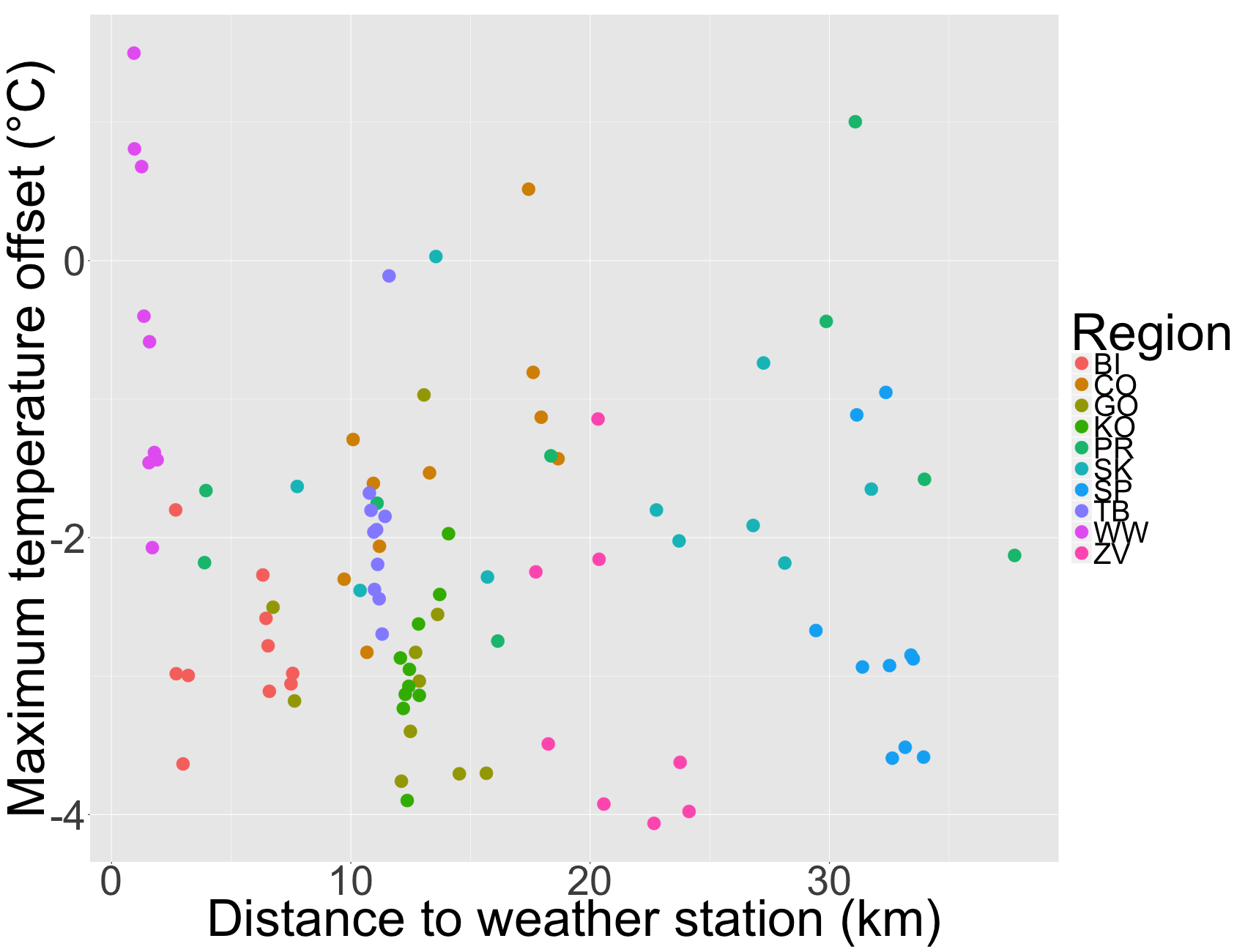

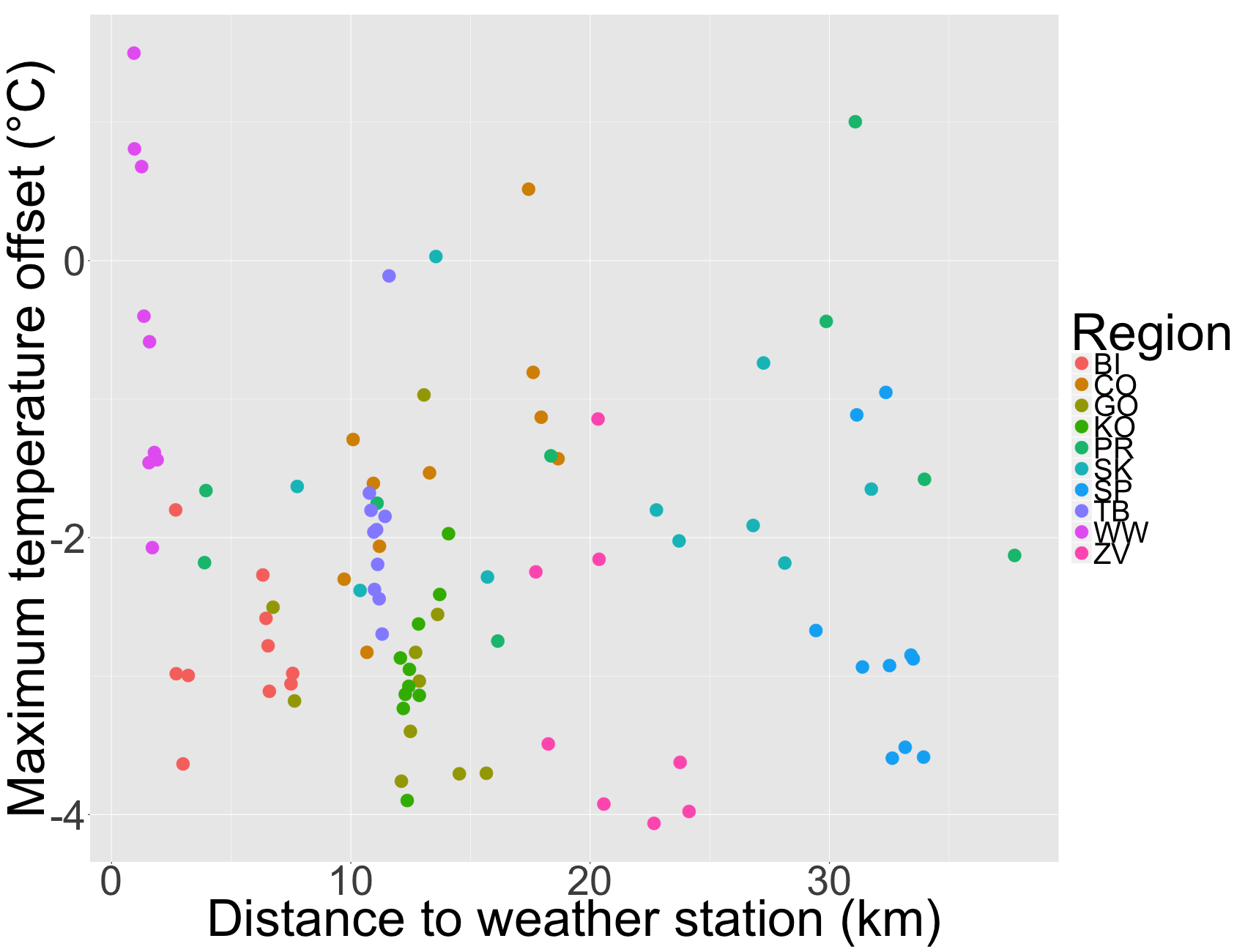


**
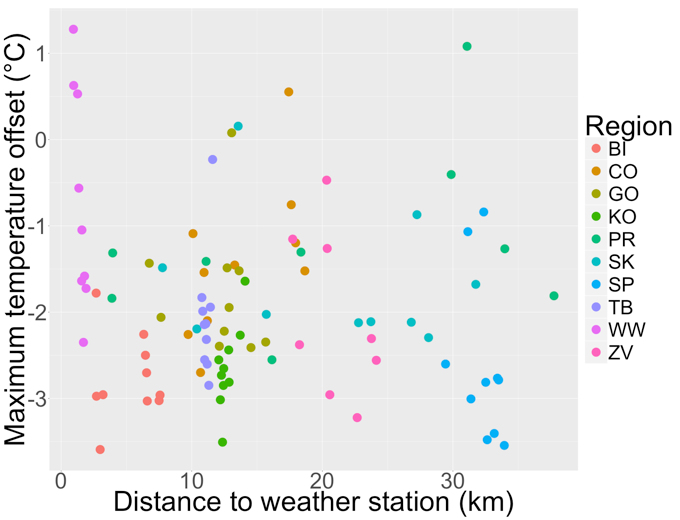

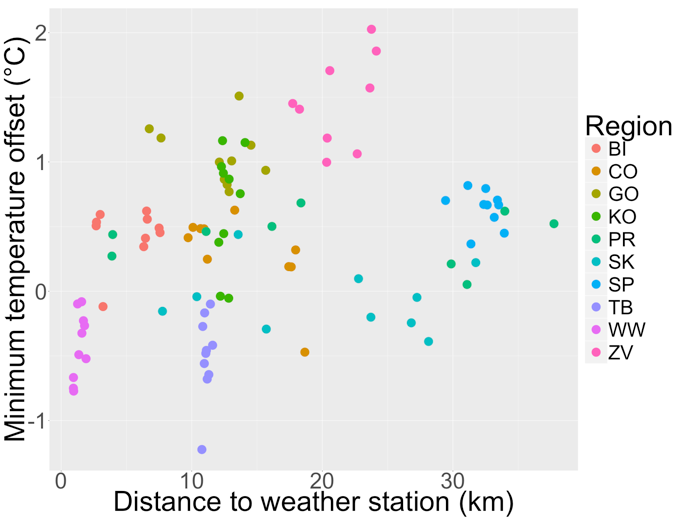
Figure S2a**: A: Scatterplot of maximum temperature values during summer and the absolute difference in elevation between each plot and the weather station to which the plot data was compared to. B: Same as A but for minimum temperature buffering during winter. C & D: Same as A & B but with the spatial distance to the weather station on the x-axis.

**
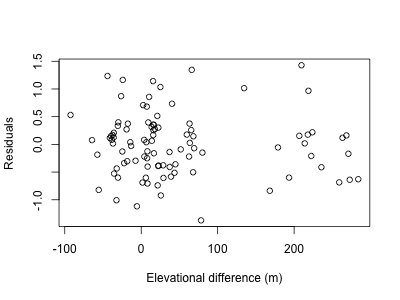
**
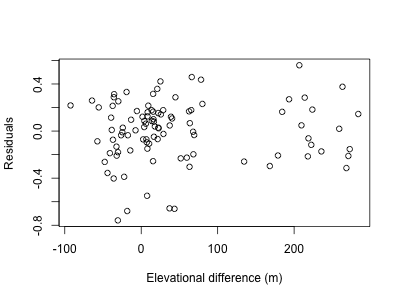


B
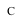

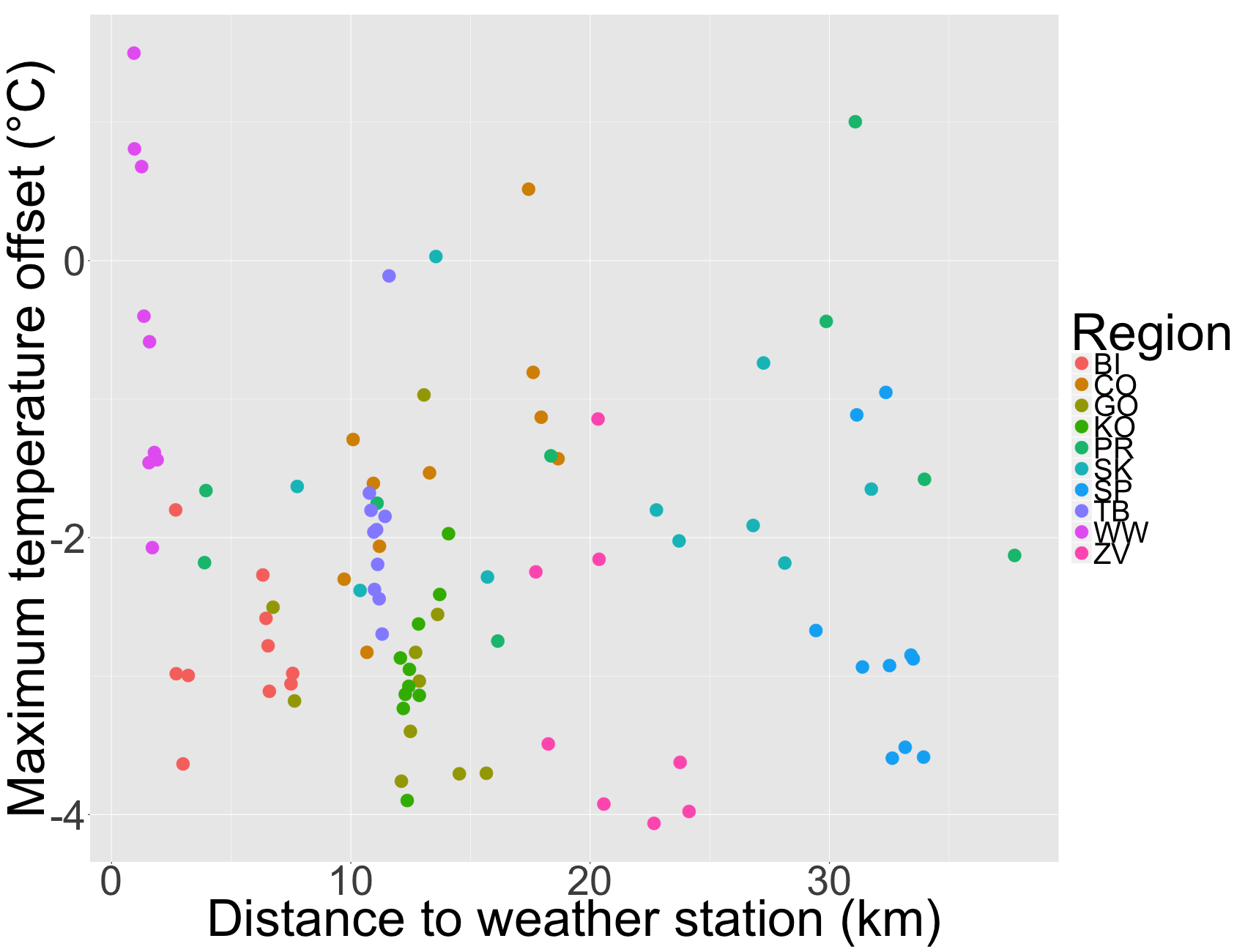

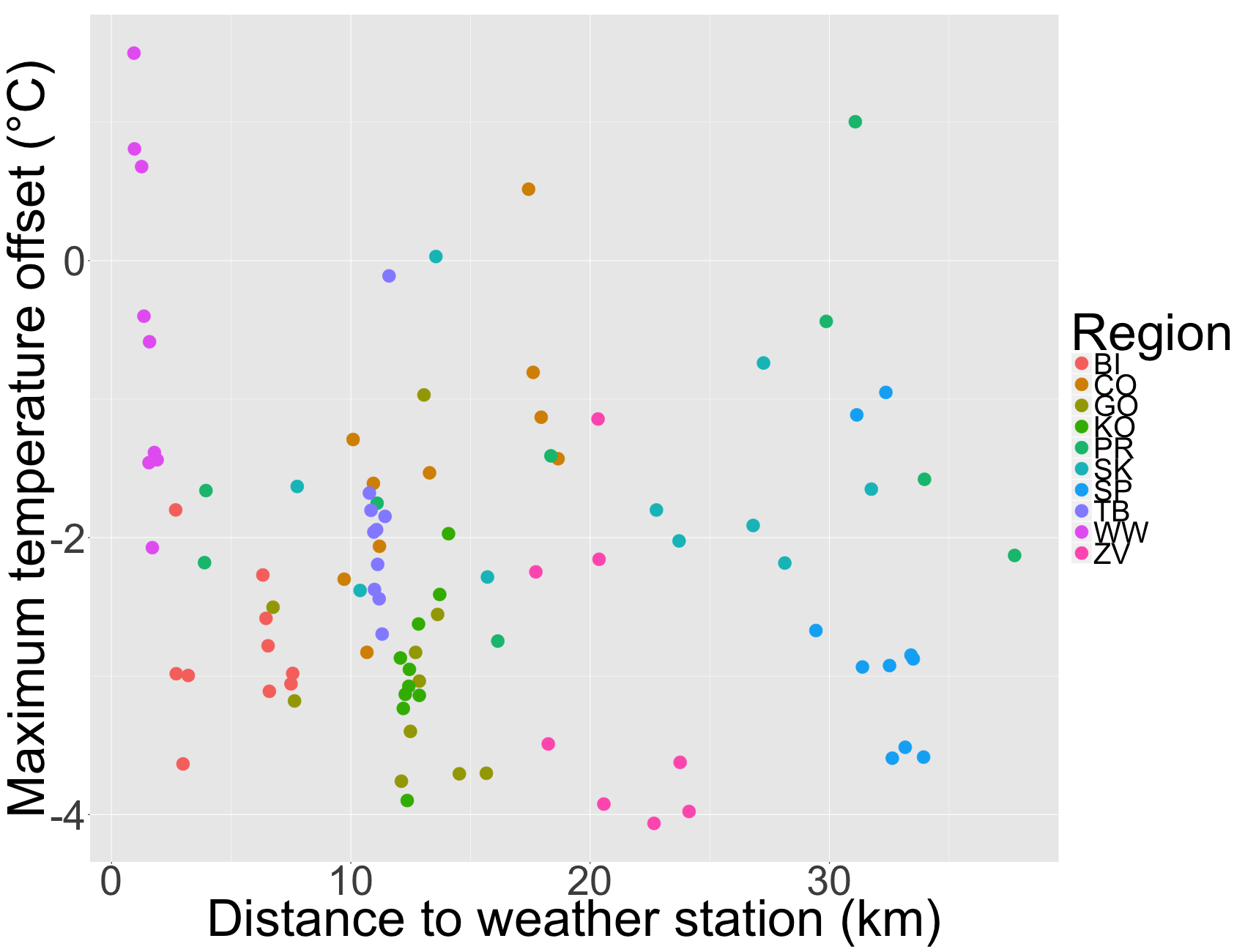


A
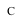

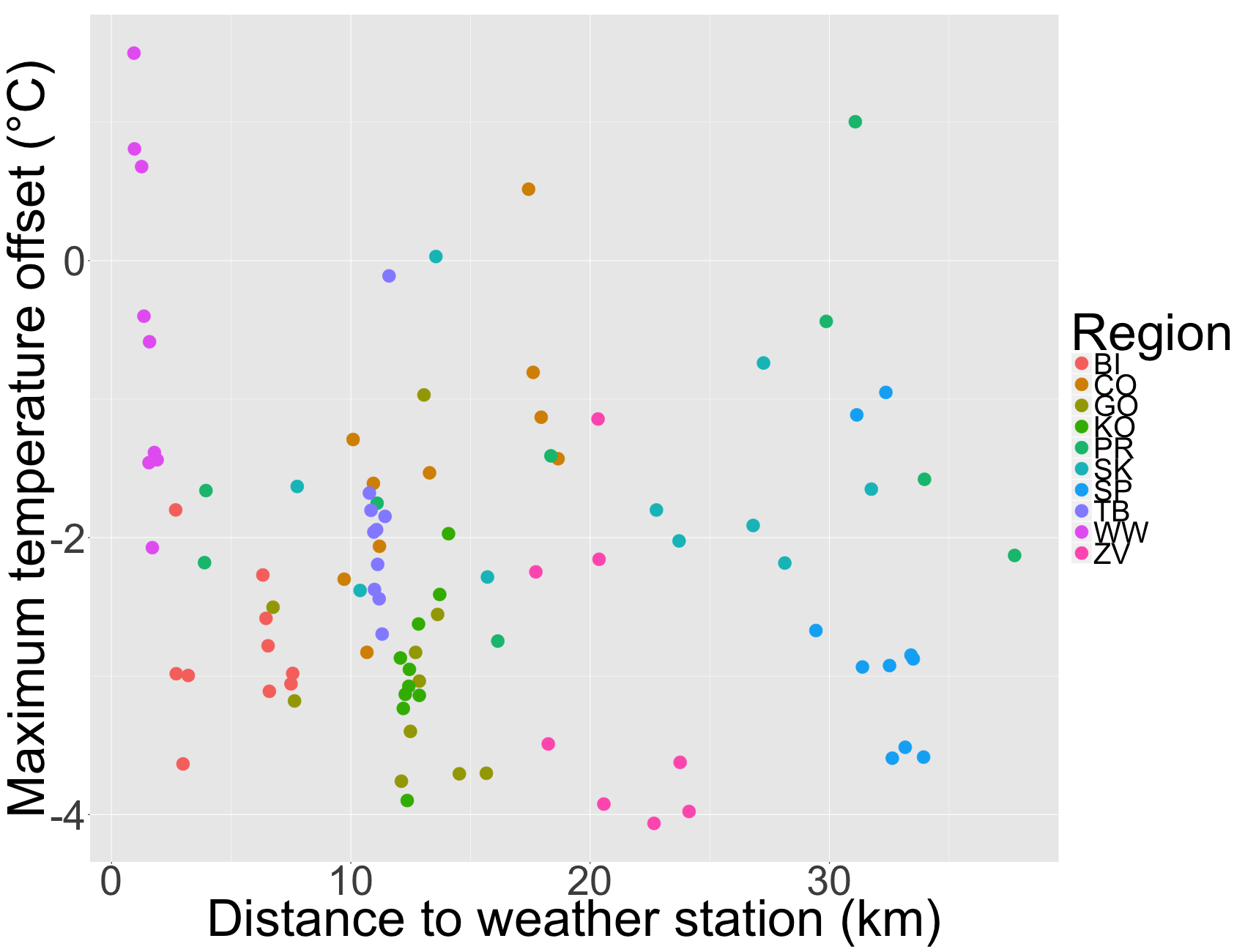

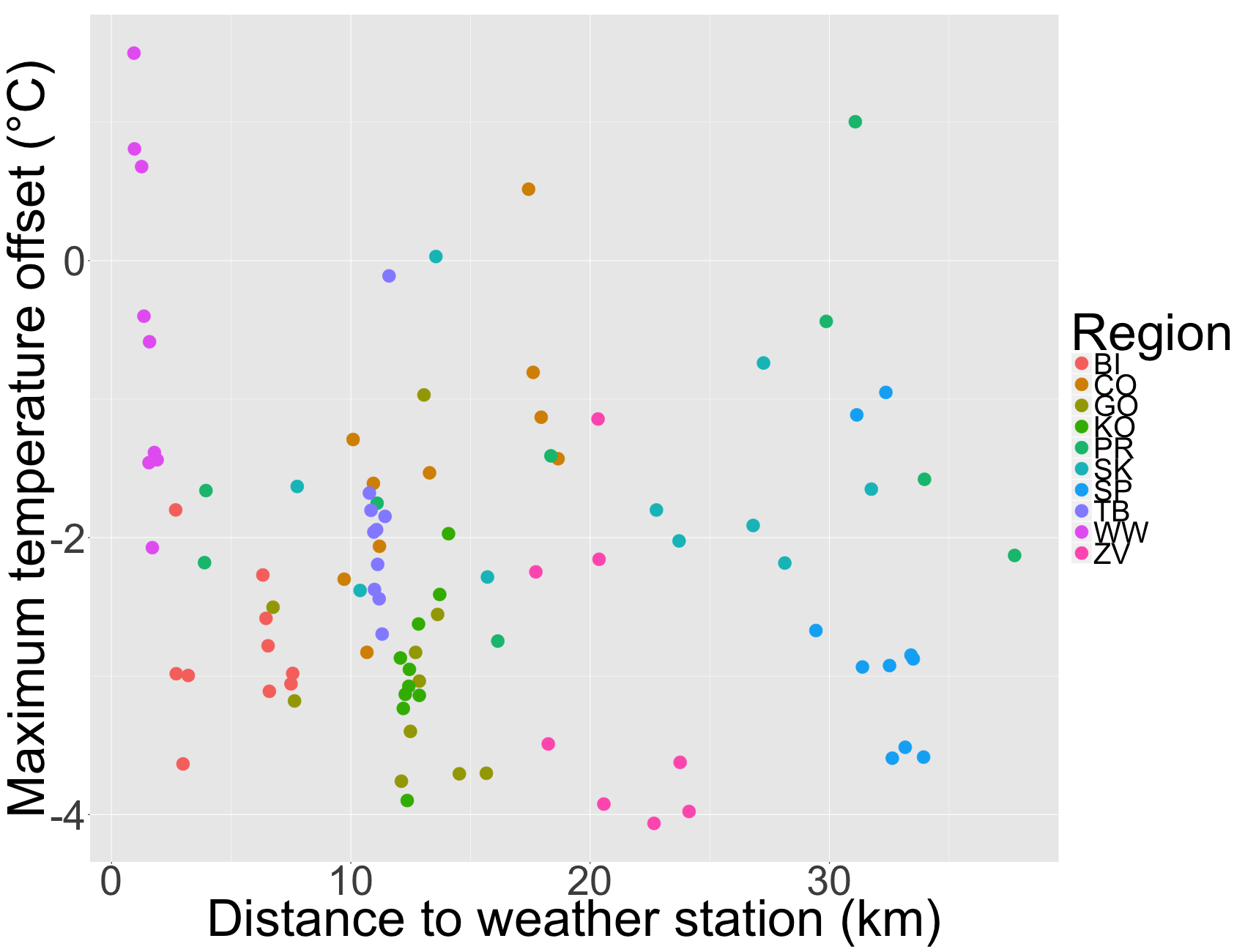


**Figure S2b**: A: Scatterplot of residuals from the GAMM for Tmax offset during summer as a function of canopy cover, and the elevational differences between each plot and the weather station. B: Same as A but considering the model for Tmin offset during winter as a function of elevation, i.e. the strongest predictor of Tmin offset during winter (Table S8).


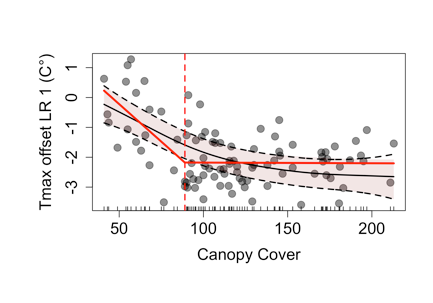

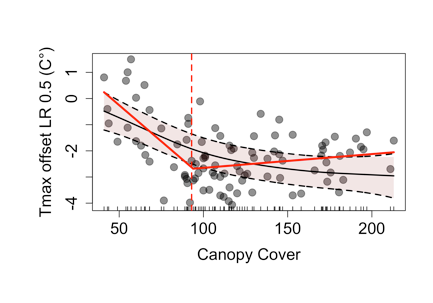

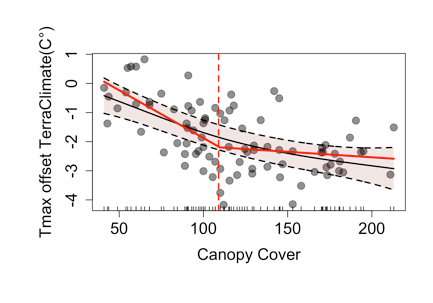
**Figure S2c**: Relationships between canopy cover and the offset of Tmax offset during summer, calculated with lapse rate of 1 °C per 100m (A), 0.5 °C per 100m (B), and using interpolated climate data from TerraClimate (Abatzoglou *et al.*, 2018) (C). Smoothed curves with 95 % confidence intervals (light red polygons) and break point analysis as described in the main text.

C
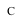

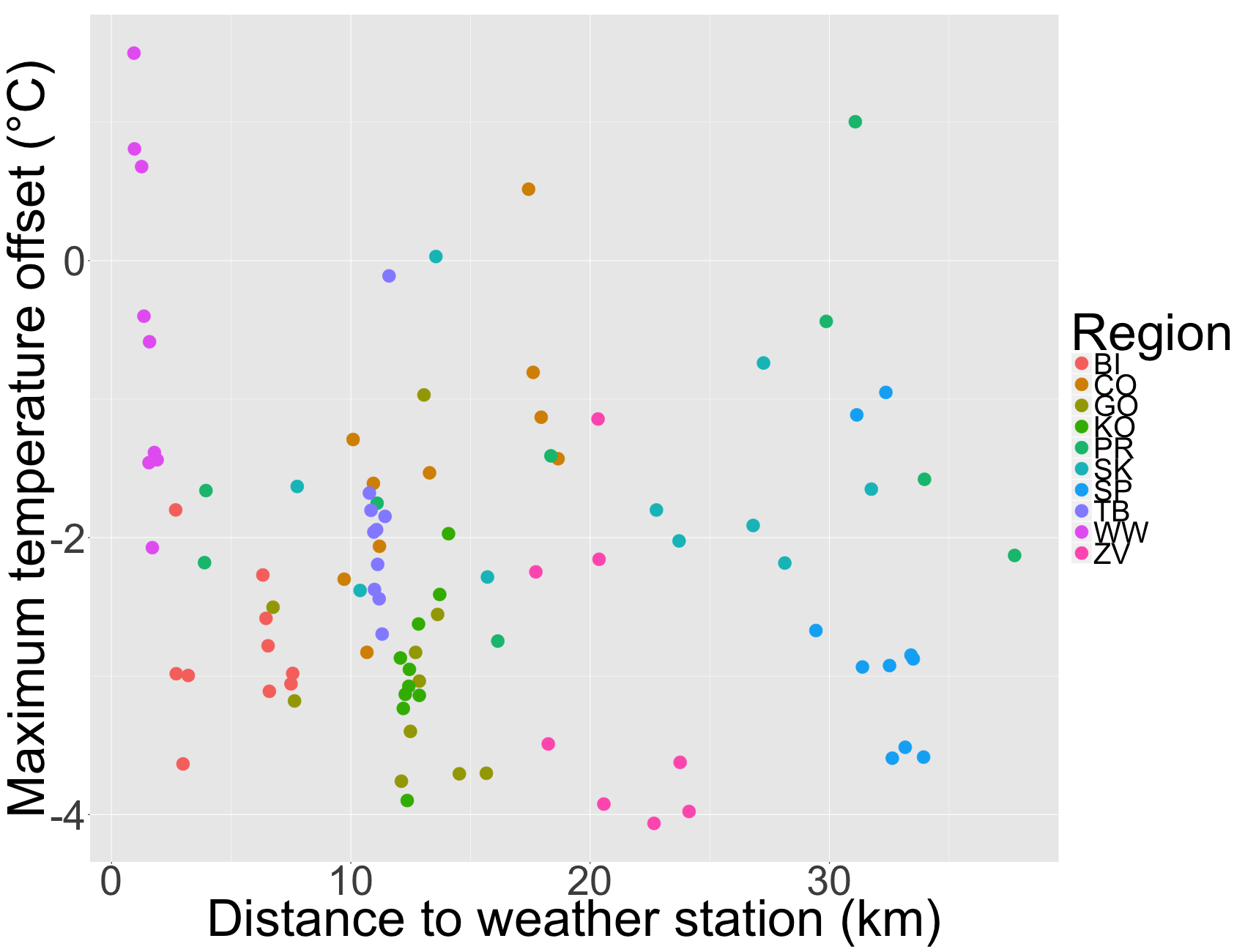

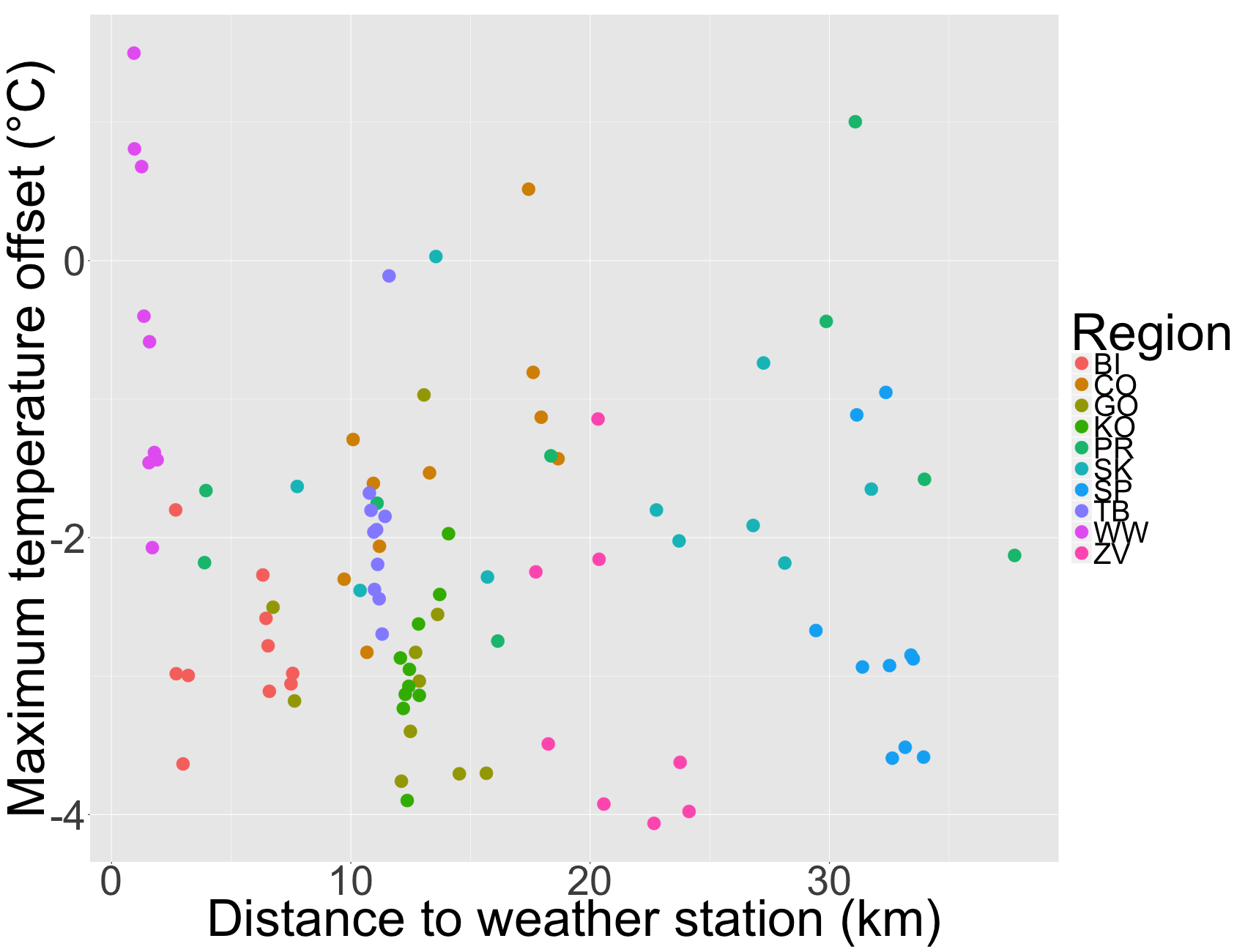


B
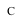

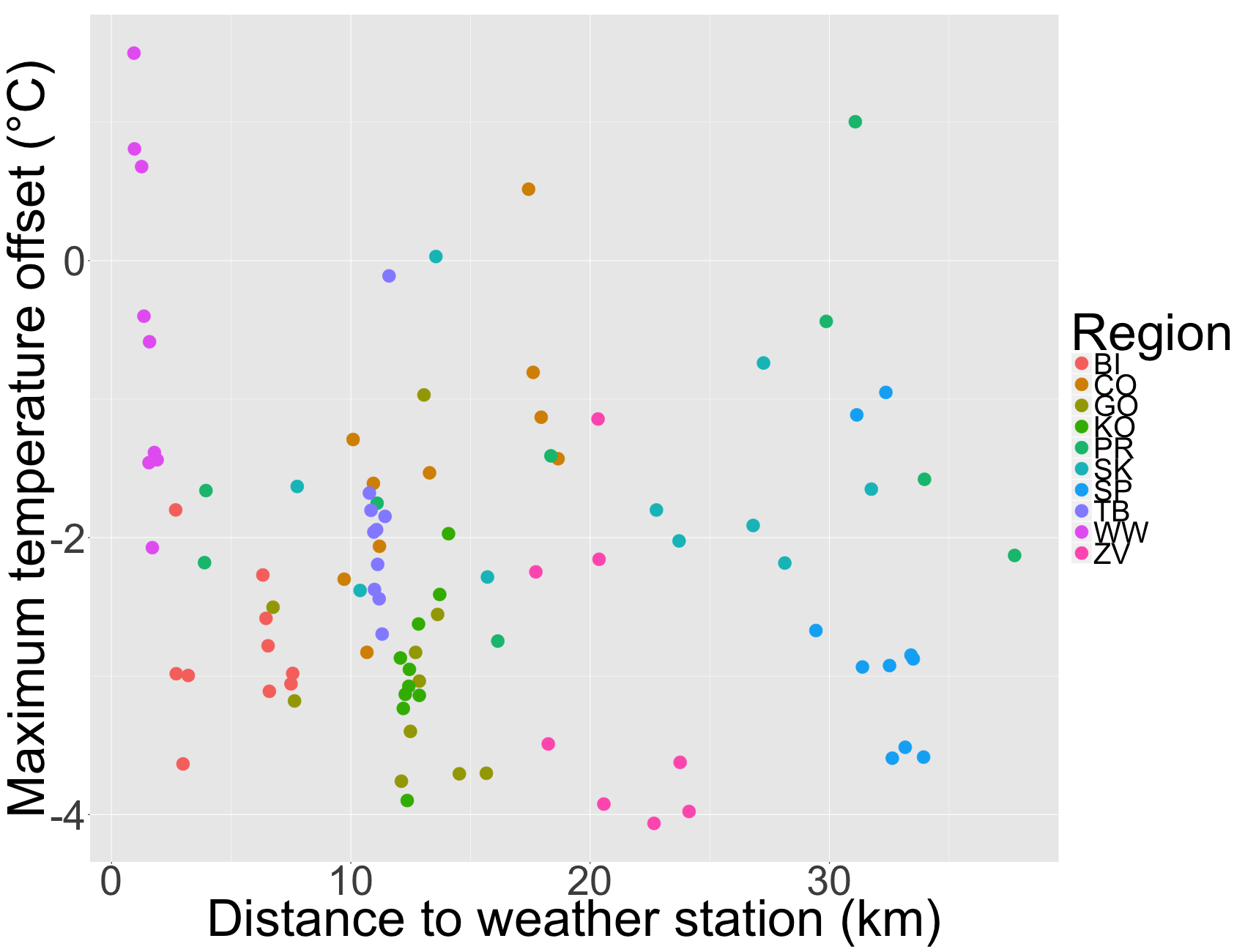

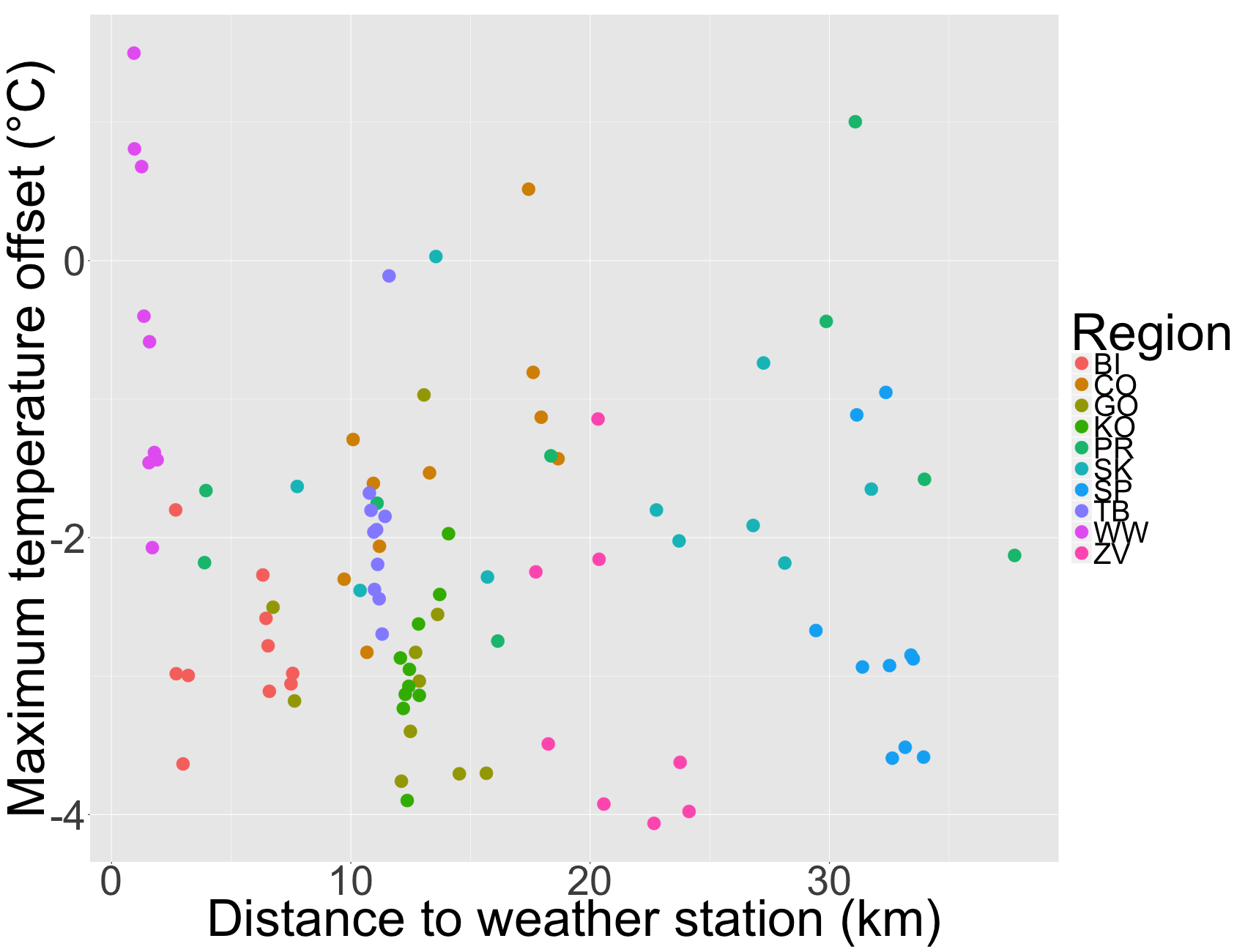


A
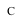

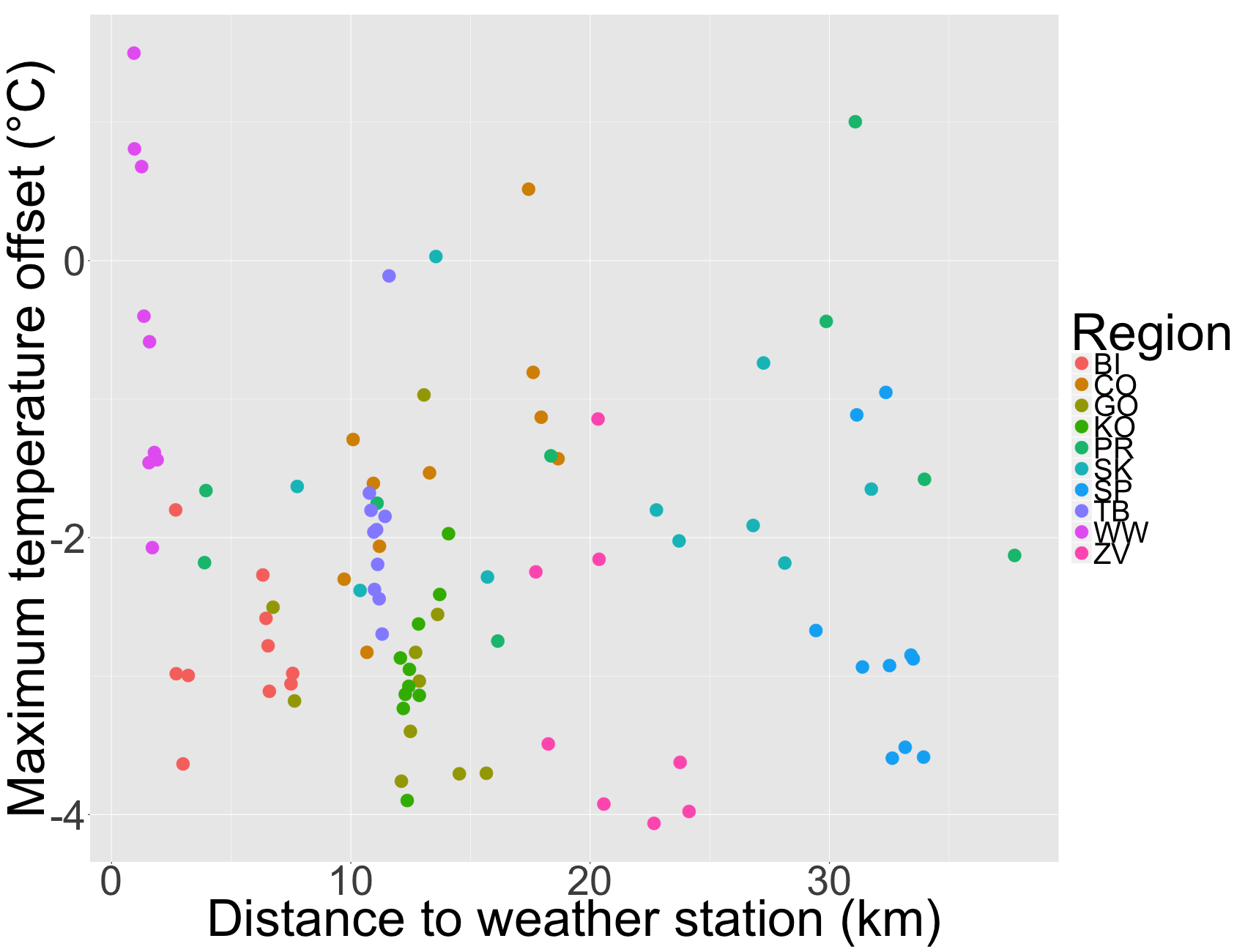

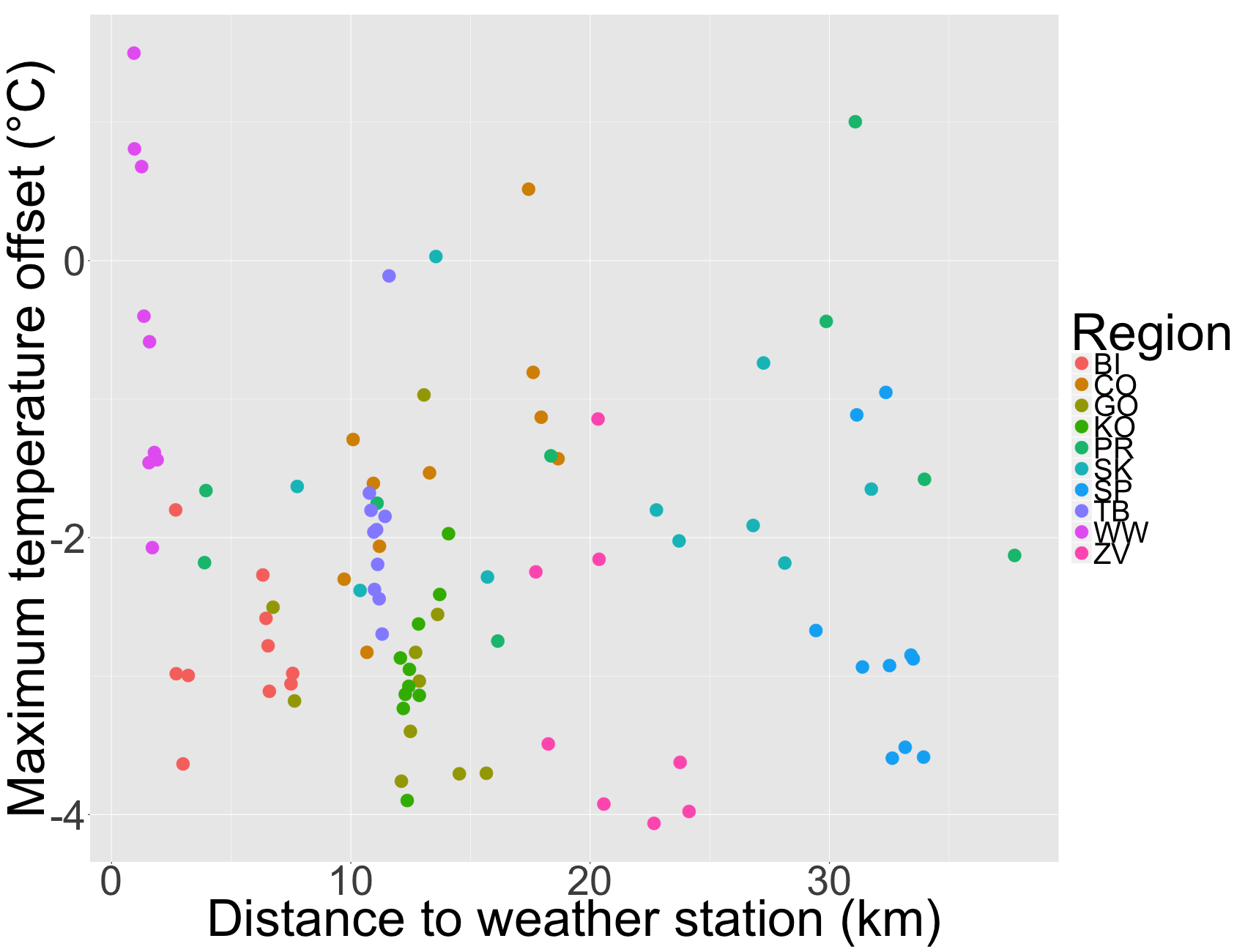


C
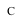

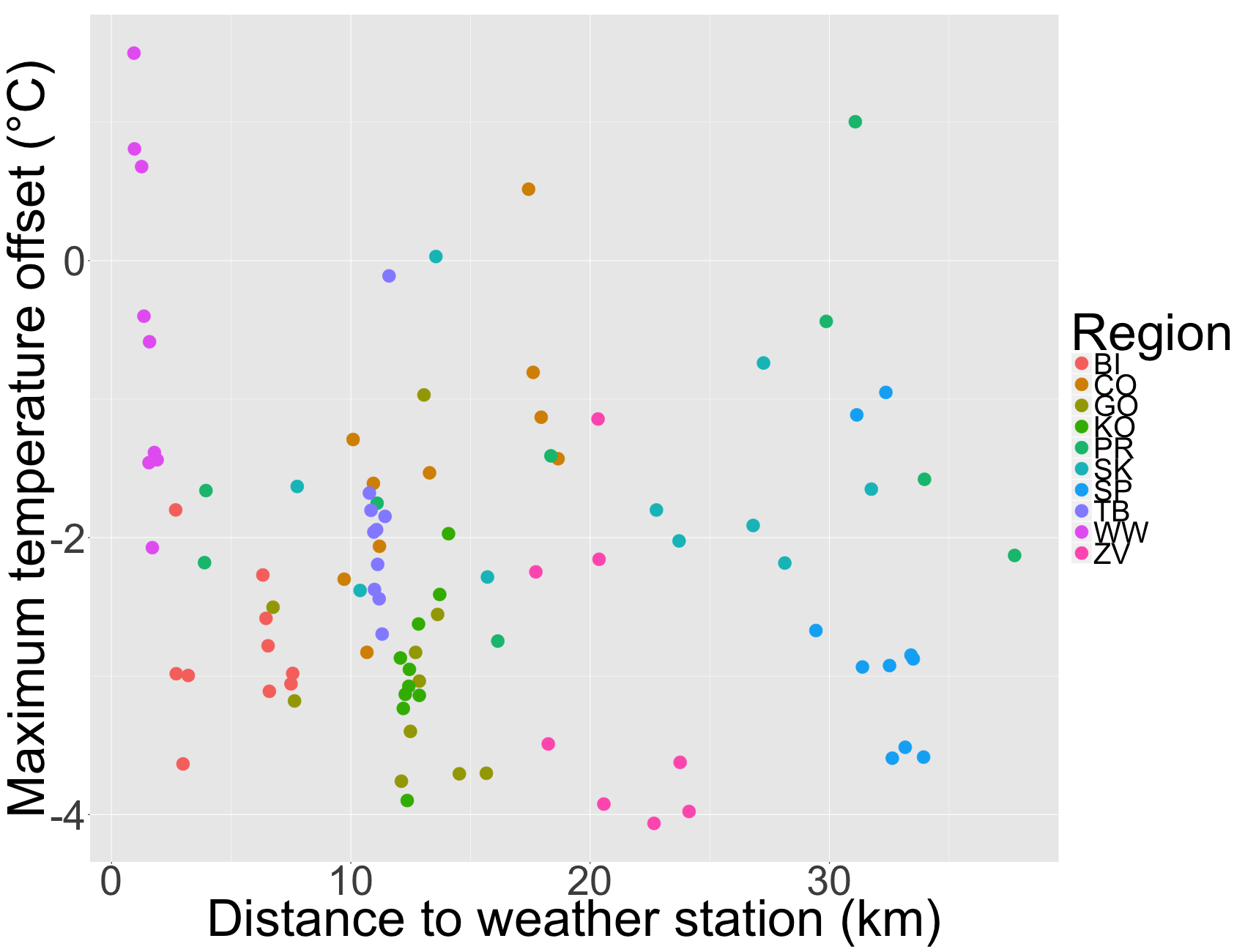

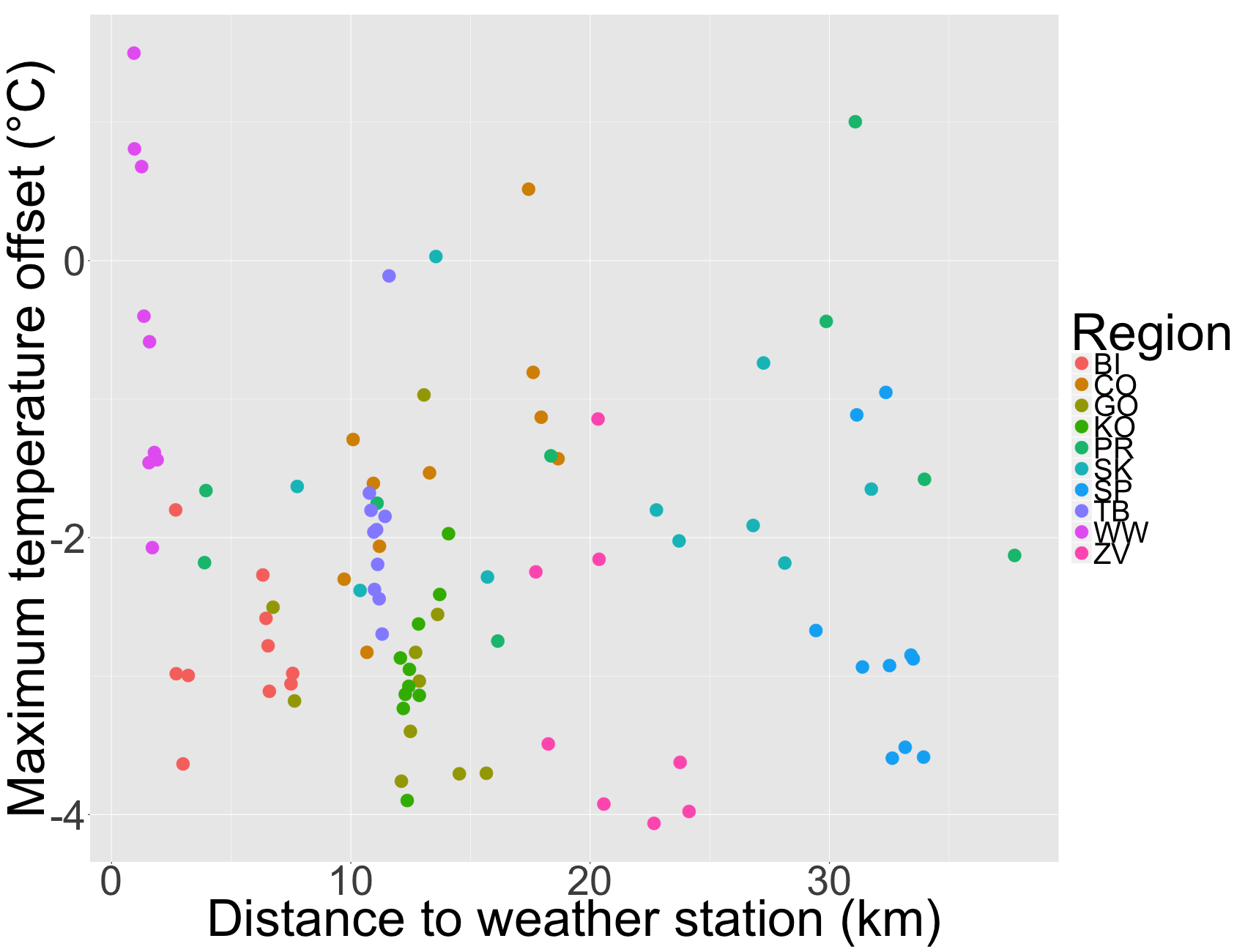


A
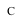

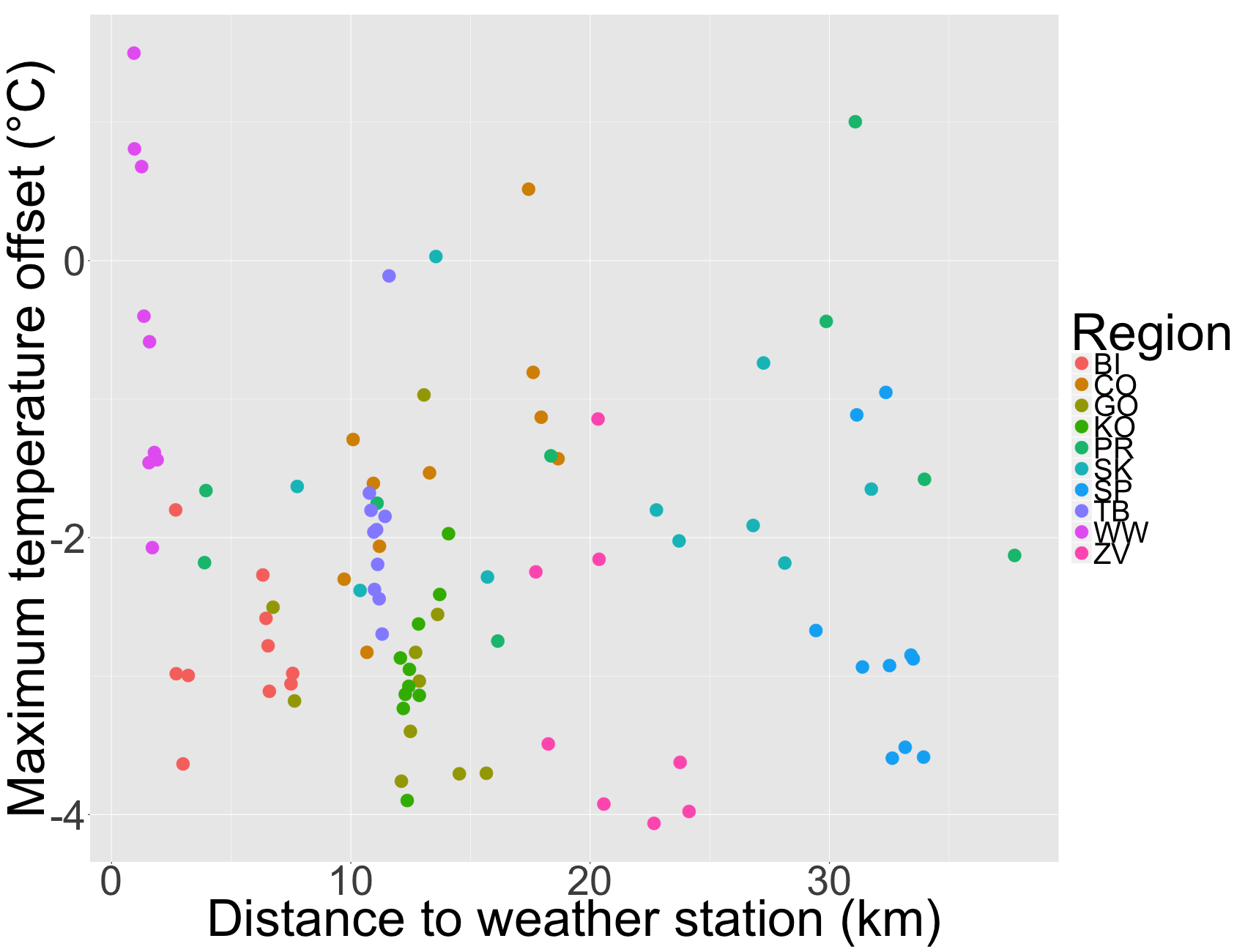

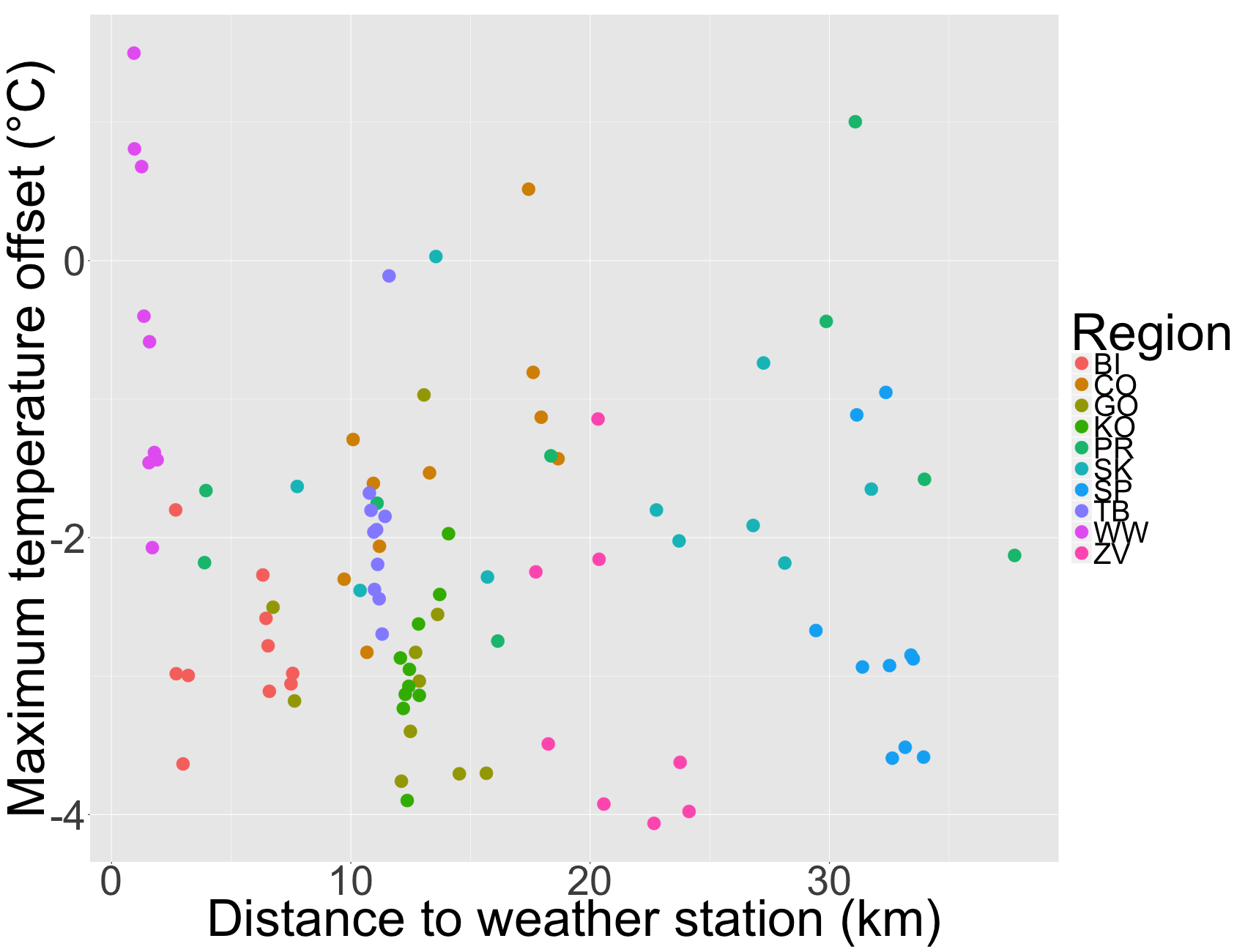


B
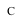

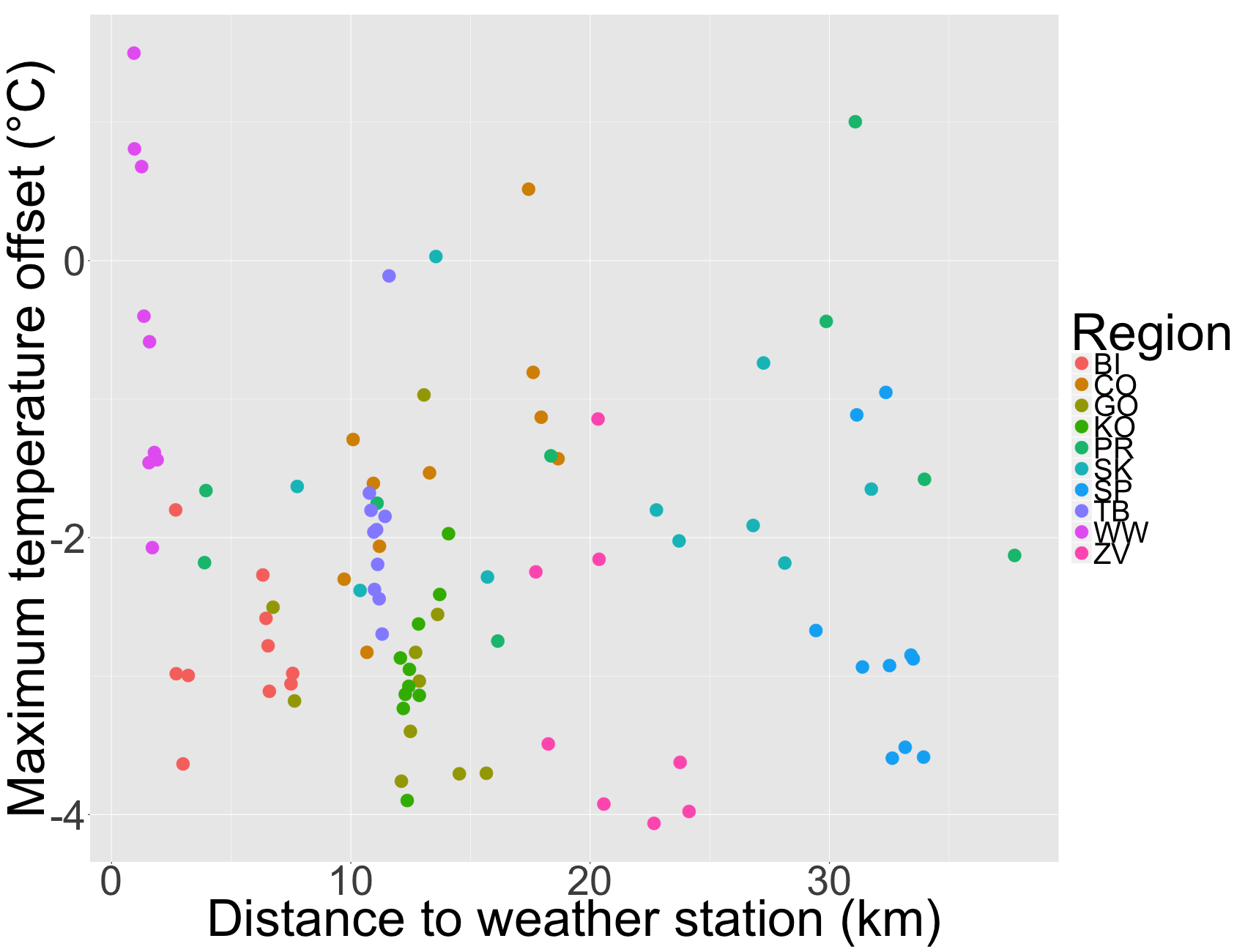

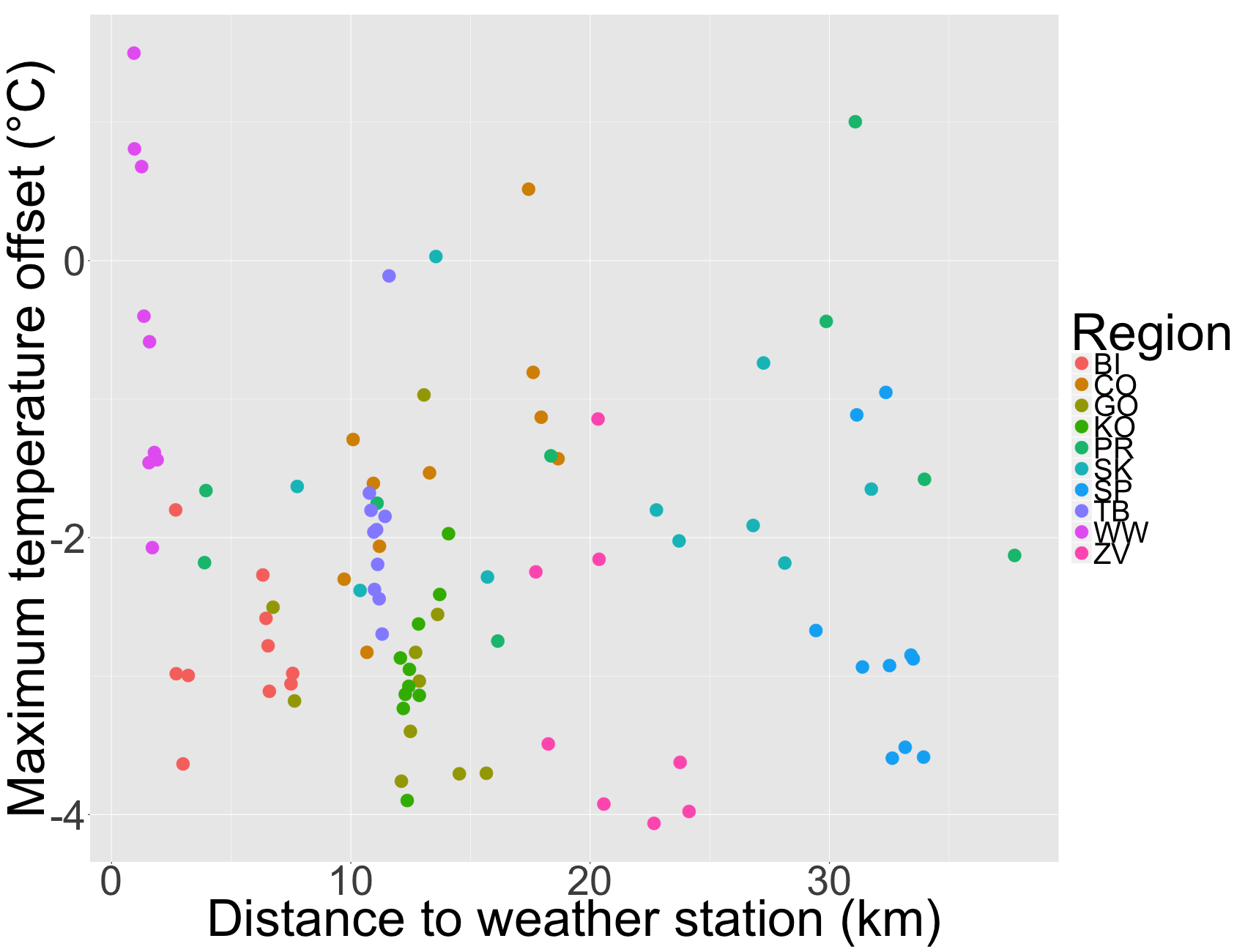


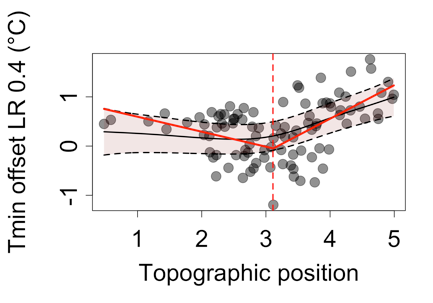

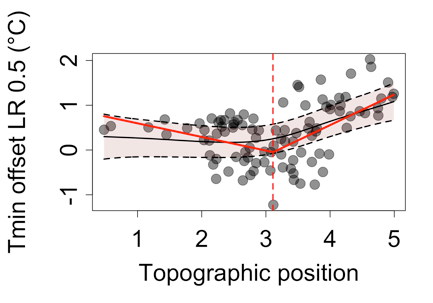

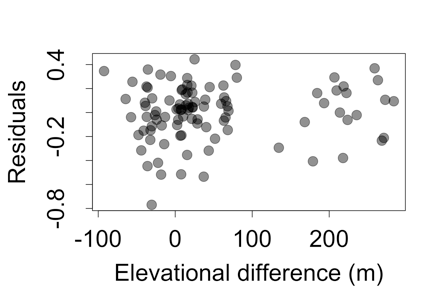


**Figure S2d**: Relationships between the relative topographic position and the offset of daily minimum temperatures during winter, calculated using a lapse rate of 0.4 °C per 100m (A) and 0.5 °C per 100m (B). C show the residuals from the GAMM model in B over the elevational differences between to the weather stations.

## Appendix S3 | Extended Canopy Cover analysis

The canopy cover values presented in the main text represent the cumulative sum of cover across all tree and shrub species and vertical layers, which means that values can exceed 100 percent due to overlaps between species. In practice, however, canopy cover is often estimated on the stand level, not species level, and the values therefore often range between 0 and 100 percent. We thus extended our analysis of canopy cover to account for this, by transforming and constraining our canopy cover values to values not exceeding 100 percent. To this end, we used the approach described by Fischer (2015), which corrects for the overlap effect between different species and vertical layers. This approach aims to correct for the exponential effect of light extinction along the vertical forest profile following the Beer-Lambert law (Campbell, 1986). The statistical relationship between the canopy cover (Fischer) and the offset of the maximum temperature during summer is significant at *p* < 0.001 and shows a threshold at 75 % (standard error 5.2) cover (Figure S9).

**
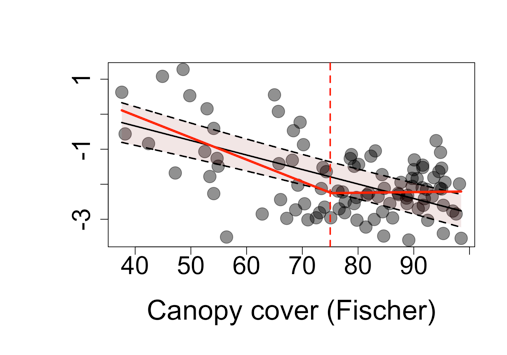
**

Tmax offset (°c)

**Figure S3**. Relationships between canopy cover following the Fischer approach, and the offset of daily maximum temperatures during summer. The light red polygons represent the 95 % confidence. The red vertical dashed line indicates the threshold at 75 %, and the solid red line shows the regression lines as calculated using piecewise regression (see text for details).

## Appendix S4 | Estimation of Crown Area

We calculated the projected crown area (CA) for each species based on the allometric relationship between CA and BHD. We used CA and DBH data from an open-access global database (Jucker *et al.*, 2016) to model CA as a function of DBH, using a linear mixed-effects log-log model. We only considered trees measured in temperate forests (n=8060). Scaling relationships between DBH and CA vary among species and are influenced by climate (Jucker *et al.*, 2016). We thus used log(CA) as dependent variable and log(DBH) as well as the mean annual temperature (MAT) and precipitation (MAP) as predictor variables, and included the term ‘functional group’, which distinguishes angiosperm and gymnosperm tree species, as a random effect. Although our plots were dominated by angiosperm species, gymnosperm tree species occurred in five plots. MAT and MAP were derived from (Karger *et al.*, 2017). The *R^2^* of this model, representing the variation explained by the fixed factors, was 0.55. We then used this model to predict CA for each of the trees measured in our plots and calculated the sum of CA per plot.

## Appendix S5 | Principal components analysis


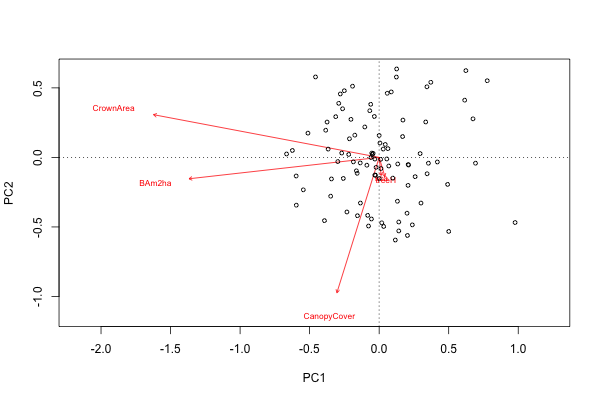


**A**


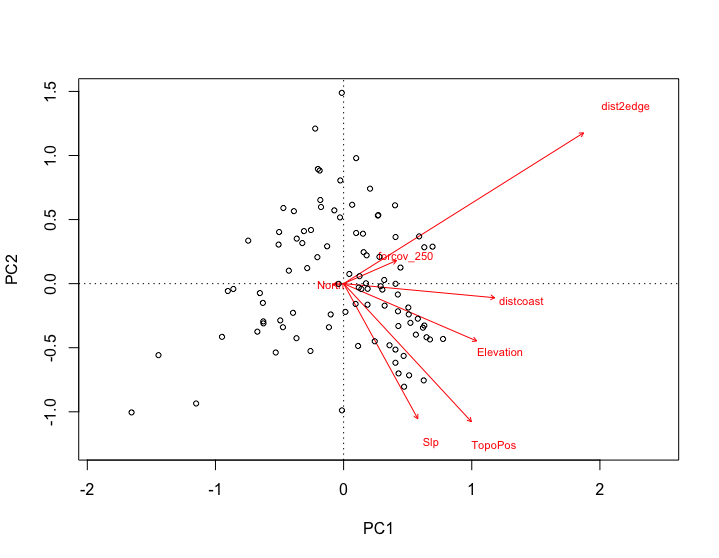


**B**


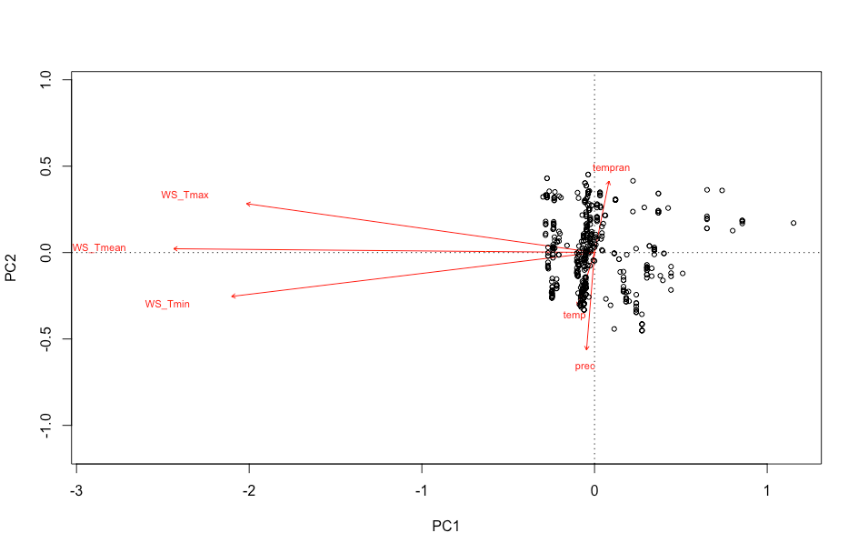


**C**

**Figure S5**. Principal components analysis plots for (A) local canopy structure and composition, (B) landscape structure and topography, and (C) the macroclimate represented by weather station records (WS_Tmin, WS_Tmean and WS_Tmax) and mean annual temperature (temp), precipitation (prec) and temperature range (tempran). The red arrows indicate the relative loadings of each variable.

## Appendix S6 | Monthly temperature offset values per region


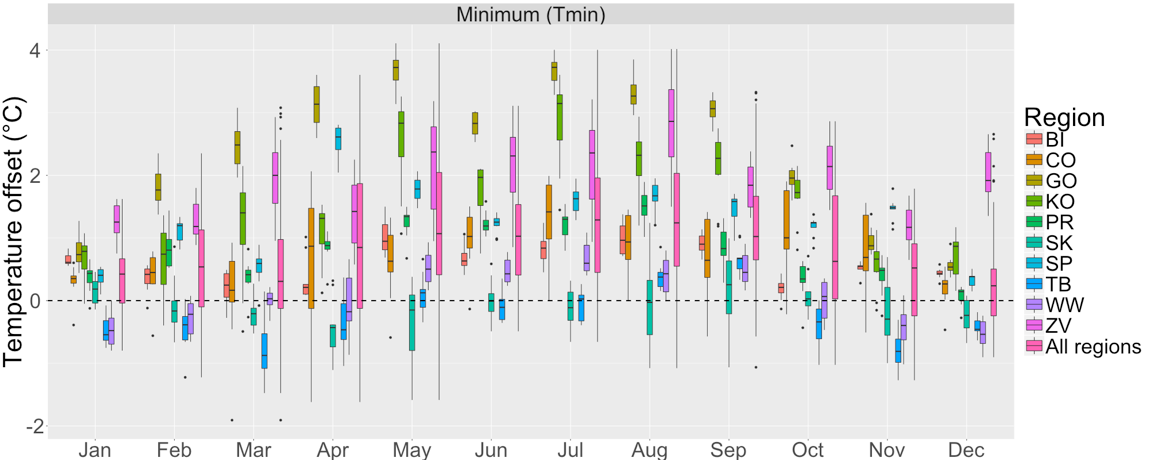


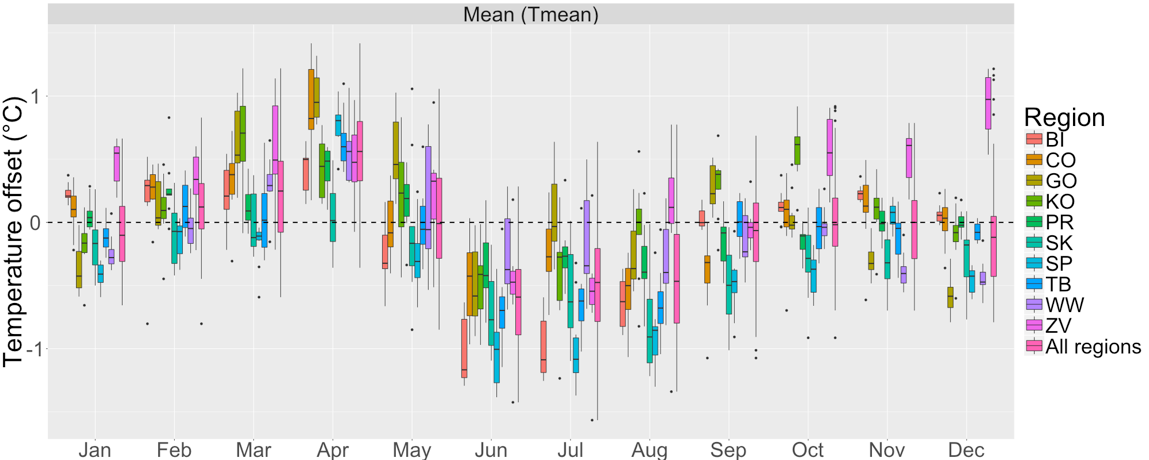


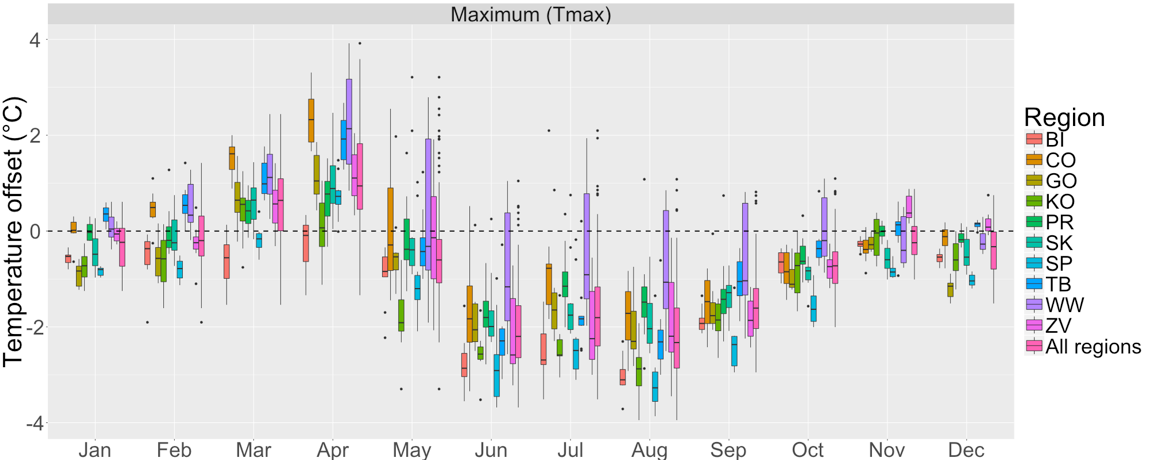


**Figure S6**. Boxplots for monthly offset values of minimum (Tmin), mean (Tmean) and maximum (Tmax) temperatures per regions and across all regions, calculated as the difference between temperatures measured inside forests minus the temperature recorded by a nearby weather station. Negative offset values thus indicate cooler conditions and positive values indicate warmer conditions inside forests.

## Appendix S7 | Offset values of absolute minimum temperatures in spring and winter


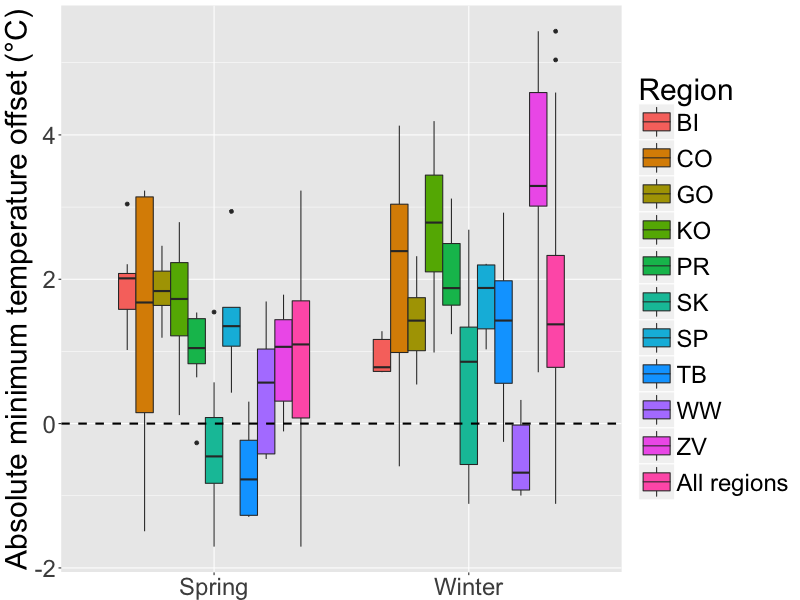


**Figure S7**. Boxplots for offset values of absolute minimum temperatures in spring and winter, calculated as the difference between temperatures measured inside forests minus the temperature recorded by a nearby weather station. Positive offset values thus indicate warmer conditions and negative values indicate cooler conditions inside forests.

**Table S7**. Individual variable effects on the absolute minimum temperature offset during winter and spring, as derived from linear mixed-effects models (LMMs). We performed χ2-tests by comparing the univariate LMM including each single predictor with a respective intercept-only model, both with ‘region’ as a random effect. Marginal *R^2^* (*R^2^m*) describes variation explained by fixed factors only; conditional *R^2^* (*R^2^c*) the variation explained by the fixed and random factors.

| Dependent variable | Predictor variable | Intercept | Estimate | Chisq | p | R2m | R2c |
| --- | --- | --- | --- | --- | --- | --- | --- |
| Offset of absolute Tmin in winter | Canopy cover | 1.561 | 0.074 | 0.374 | 0.541 | 0.003 | 0.524 |
|  | Canopy openness | 1.561 | -0.136 | 1.529 | 0.216 | 0.009 | 0.515 |
|  | Basal area | 1.558 | 0.234 | 4.776 | 0.029 | 0.029 | 0.513 |
|  | Crown area | 1.561 | -0.052 | 0.206 | 0.650 | 0.001 | 0.523 |
|  | Tree height | 1.557 | 0.241 | 3.050 | 0.081 | 0.030 | 0.504 |
|  | Shade casting ability | 1.560 | -0.087 | 0.368 | 0.544 | 0.004 | 0.537 |
|  | Forest cover | 1.565 | 0.240 | 2.617 | 0.106 | 0.028 | 0.550 |
|  | Distance to forest edge | 1.562 | 0.138 | 1.038 | 0.308 | 0.009 | 0.535 |
|  | Northness | 1.561 | 0.033 | 0.107 | 0.744 | 0.001 | 0.521 |
|  | Slope | 1.559 | 0.149 | 1.179 | 0.278 | 0.011 | 0.500 |
|  | Elevation | 1.551 | 0.819 | 8.395 | 0.004 | 0.277 | 0.626 |
|  | Topographic position | 1.561 | 0.619 | 15.252 | 0.000 | 0.168 | 0.640 |
|  | Distance to coast | 1.552 | 0.634 | 5.110 | 0.024 | 0.199 | 0.520 |
| Offset of absolute Tmin in spring | Canopy cover | 0.957 | 0.270 | 6.106 | 0.013 | 0.045 | 0.570 |
|  | Canopy openness | 0.958 | -0.225 | 5.352 | 0.021 | 0.034 | 0.527 |
|  | Basal area | 0.949 | 0.246 | 6.369 | 0.012 | 0.041 | 0.520 |
|  | Crown area | 0.954 | -0.205 | 3.947 | 0.047 | 0.027 | 0.532 |
|  | Tree height | 0.945 | 0.198 | 2.439 | 0.118 | 0.027 | 0.496 |
|  | Shade casting ability | 0.955 | -0.077 | 0.250 | 0.617 | 0.004 | 0.532 |
|  | Forest cover | 0.960 | 0.093 | 0.487 | 0.485 | 0.006 | 0.488 |
|  | Distance to forest edge | 0.959 | 0.249 | 4.359 | 0.037 | 0.044 | 0.479 |
|  | Northness | 0.956 | 0.125 | 1.870 | 0.171 | 0.010 | 0.509 |
|  | Slope | 0.955 | 0.138 | 1.162 | 0.281 | 0.013 | 0.510 |
|  | Elevation | 0.960 | 0.432 | 2.213 | 0.137 | 0.105 | 0.594 |
|  | Topographic position | 0.960 | 0.328 | 5.112 | 0.024 | 0.073 | 0.514 |
|  | Distance to coast | 0.953 | 0.337 | 1.765 | 0.184 | 0.075 | 0.509 |

## Appendix S8 | Extended results from variation partitioning

**Table S8**. Results from variation partitioning separating the independent share of explained variation (*R^2^m*) for each variable group, i.e., canopy and landscape, from the shared amount of explained variation (canopy ∩ landscape). Marginal *R^2^* (*R^2^m*) describes variation explained by fixed factors only; conditional *R^2^* (*R^2^c*) the variation explained by the fixed and random factors.

| **Temperature offset** | **Season/Period** | **Canopy** | **Landscape** | **Canopy ∩ Landscape** | **Total *R^2^m*** | **Total *R^2^c*** |
| --- | --- | --- | --- | --- | --- | --- |
| T_min_ | Spring | 0.01 | 0.19 | 0.00 | 0.20 | 0.83 |
|  | Summer | 0.01 | 0.29 | -0.01 | 0.29 | 0.87 |
|  | Autumn | 0.01 | 0.26 | 0.00 | 0.28 | 0.81 |
|  | Winter | 0.02 | 0.40 | -0.01 | 0.41 | 0.83 |
|  | All year | 0.01 | 0.29 | 0.00 | 0.30 | 0.85 |
| T_max_ | Spring | 0.11 | 0.07 | 0.02 | 0.18 | 0.58 |
|  | Summer | 0.22 | 0.15 | 0.04 | 0.41 | 0.73 |
|  | Autumn | 0.09 | 0.01 | 0.03 | 0.13 | 0.63 |
|  | Winter | 0.00 | 0.03 | 0.00 | 0.03 | 0.65 |
|  | All year | 0.10 | 0.13 | 0.03 | 0.27 | 0.71 |
| T_mean_ | Spring | 0.03 | 0.06 | 0.01 | 0.09 | 0.38 |
|  | Summer | 0.14 | 0.01 | 0.02 | 0.17 | 0.52 |
|  | Autumn | 0.00 | 0.51 | 0.01 | 0.53 | 0.55 |
|  | Winter | 0.00 | 0.21 | 0.00 | 0.21 | 0.56 |
|  | All year | 0.02 | 0.18 | 0.03 | 0.23 | 0.39 |

**Figure S8**. Results from variation partitioning separating the independent (grey area in top inset) and joint share of explained variation (*R^2^m*) among three variable groups, i.e. (1) local forest canopy characteristics; (2) landscape structure and topography, and (3) the macroclimate. The marginal *R^2^* (*R^2^m*) describes variation explained by fixed factors only (i.e., first and second PCA axes per variable group); conditional *R^2^* (*R^2^c*) the variation explained by the fixed factors and the random factor ‘region’.

## Appendix S9 | Individual variable effects on the temperature offset

**Table S9**. Individual variable effects on the temperature offset for each season and the entire year, as derived from linear mixed-effects models (LMMs). All variables were standardised (mean of zero and standard deviation of one) prior to analysis so that estimates can be interpreted as relative effect sizes. We performed χ2-tests by comparing the univariate LMM including each single predictor with a respective intercept-only model, both with ‘region’ as a random effect. Marginal *R^2^* (*R^2^m*) describes variation explained by fixed factors only; conditional *R^2^* (*R^2^c*) the variation explained by the fixed and random factors.

| **Spring** | |  |  |  | | |  | | |  | | |  | |
| --- | --- | --- | --- | --- | --- | --- | --- | --- | --- | --- | --- | --- | --- | --- |
| Dependent variable | Predictor variable | | | | Intercept | Estimate | | Chisq | p | | R2m | R2c | |  |
| Offset of Tmin | Canopy cover | | | | 0.941 | 0.101 | | 2.327 | 0.127 | | 0.007 | 0.832 | |  |
|  | Canopy openness | | | | 0.938 | -0.167 | | 8.526 | 0.003 | | 0.019 | 0.840 | |  |
|  | Basal area | | | | 0.939 | 0.102 | | 2.910 | 0.088 | | 0.007 | 0.823 | |  |
|  | Crown area | | | | 0.939 | 0.084 | | 1.021 | 0.312 | | 0.004 | 0.821 | |  |
|  | Tree height | | | | 0.940 | -0.037 | | 0.371 | 0.542 | | 0.001 | 0.822 | |  |
|  | Shade casting ability | | | | 0.941 | 0.009 | | 0.024 | 0.877 | | 0.000 | 0.819 | |  |
|  | Forest cover | | | | 0.944 | 0.078 | | 0.782 | 0.377 | | 0.004 | 0.818 | |  |
|  | Distance to forest edge | | | | 0.946 | 0.135 | | 3.535 | 0.060 | | 0.013 | 0.821 | |  |
|  | Northness | | | | 0.941 | 0.007 | | 0.017 | 0.896 | | 0.000 | 0.820 | |  |
|  | Slope | | | | 0.939 | 0.034 | | 0.206 | 0.650 | | 0.001 | 0.820 | |  |
|  | Elevation | | | | 0.918 | 1.223 | | 21.919 | 0.000 | | 0.530 | 0.932 | |  |
|  | Topographic position | | | | 0.941 | 0.476 | | 30.574 | 0.000 | | 0.211 | 0.827 | |  |
|  | Distance to coast | | | | 0.929 | 0.481 | | 2.686 | 0.101 | | 0.166 | 0.822 | |  |
| Offset of Tmax | Canopy cover | | | | 0.355 | -0.335 | | 12.150 | 0.000 | | 0.093 | 0.590 | |  |
|  | Canopy openness | | | | 0.365 | 0.429 | | 27.451 | 0.000 | | 0.176 | 0.576 | |  |
|  | Basal area | | | | 0.358 | -0.166 | | 3.548 | 0.060 | | 0.026 | 0.430 | |  |
|  | Crown area | | | | 0.359 | -0.199 | | 3.002 | 0.083 | | 0.037 | 0.410 | |  |
|  | Tree height | | | | 0.359 | 0.244 | | 7.738 | 0.005 | | 0.061 | 0.463 | |  |
|  | Shade casting ability | | | | 0.360 | -0.272 | | 7.030 | 0.008 | | 0.076 | 0.444 | |  |
|  | Forest cover | | | | 0.363 | 0.171 | | 1.811 | 0.178 | | 0.027 | 0.474 | |  |
|  | Distance to forest edge | | | | 0.353 | -0.110 | | 1.070 | 0.301 | | 0.012 | 0.443 | |  |
|  | Northness | | | | 0.360 | -0.325 | | 18.740 | 0.000 | | 0.110 | 0.527 | |  |
|  | Slope | | | | 0.348 | 0.233 | | 4.751 | 0.029 | | 0.055 | 0.450 | |  |
|  | Elevation | | | | 0.363 | -0.333 | | 2.594 | 0.107 | | 0.102 | 0.501 | |  |
|  | Topographic position | | | | 0.357 | -0.041 | | 0.104 | 0.747 | | 0.002 | 0.430 | |  |
|  | Distance to coast | | | | 0.365 | -0.352 | | 3.530 | 0.060 | | 0.126 | 0.436 | |  |
| Offset of Tmean | Canopy cover | | | | 0.295 | -0.062 | | 3.365 | 0.067 | | 0.034 | 0.376 | |  |
|  | Canopy openness | | | | 0.297 | 0.050 | | 2.731 | 0.098 | | 0.023 | 0.344 | |  |
|  | Basal area | | | | 0.295 | -0.008 | | 0.055 | 0.815 | | 0.001 | 0.327 | |  |
|  | Crown area | | | | 0.295 | -0.017 | | 0.164 | 0.685 | | 0.002 | 0.336 | |  |
|  | Tree height | | | | 0.296 | 0.054 | | 3.001 | 0.083 | | 0.028 | 0.326 | |  |
|  | Shade casting ability | | | | 0.296 | -0.058 | | 2.095 | 0.148 | | 0.029 | 0.413 | |  |
|  | Forest cover | | | | 0.298 | 0.071 | | 3.167 | 0.075 | | 0.047 | 0.327 | |  |
|  | Distance to forest edge | | | | 0.295 | 0.014 | | 0.194 | 0.659 | | 0.002 | 0.315 | |  |
|  | Northness | | | | 0.296 | -0.113 | | 17.972 | 0.000 | | 0.122 | 0.456 | |  |
|  | Slope | | | | 0.292 | 0.096 | | 6.921 | 0.009 | | 0.088 | 0.358 | |  |
|  | Elevation | | | | 0.292 | 0.127 | | 5.682 | 0.017 | | 0.152 | 0.343 | |  |
|  | Topographic position | | | | 0.292 | 0.204 | | 29.728 | 0.000 | | 0.400 | 0.406 | |  |
|  | Distance to coast | | | | 0.293 | 0.082 | | 1.954 | 0.162 | | 0.063 | 0.352 | |  |

**Table S9**. Contiuned.

| **Summer** | |  |  | |  | |  | | |  | | |  |
| --- | --- | --- | --- | --- | --- | --- | --- | --- | --- | --- | --- | --- | --- |
| Dependent variable | Predictor variable | | | Intercept | | Estimate | | Chisq | p | | R2m | R2c |  |
| Offset of Tmin | Canopy cover | | | 1.318 | | 0.040 | | 0.618 | 0.432 | | 0.001 | 0.875 |  |
|  | Canopy openness | | | 1.317 | | -0.104 | | 5.216 | 0.022 | | 0.008 | 0.881 |  |
|  | Basal area | | | 1.317 | | 0.055 | | 1.516 | 0.218 | | 0.002 | 0.873 |  |
|  | Crown area | | | 1.317 | | 0.061 | | 1.061 | 0.303 | | 0.003 | 0.872 |  |
|  | Tree height | | | 1.318 | | 0.021 | | 0.194 | 0.659 | | 0.000 | 0.873 |  |
|  | Shade casting ability | | | 1.318 | | -0.005 | | 0.003 | 0.956 | | 0.000 | 0.873 |  |
|  | Forest cover | | | 1.320 | | 0.058 | | 0.823 | 0.364 | | 0.003 | 0.871 |  |
|  | Distance to forest edge | | | 1.319 | | 0.118 | | 4.379 | 0.036 | | 0.011 | 0.874 |  |
|  | Northness | | | 1.318 | | 0.010 | | 0.052 | 0.819 | | 0.000 | 0.873 |  |
|  | Slope | | | 1.318 | | -0.008 | | 0.015 | 0.901 | | 0.000 | 0.873 |  |
|  | Elevation | | | 1.299 | | 1.124 | | 29.195 | 0.000 | | 0.570 | 0.946 |  |
|  | Topographic position | | | 1.318 | | 0.364 | | 29.421 | 0.000 | | 0.142 | 0.870 |  |
|  | Distance to coast | | | 1.306 | | 0.558 | | 4.128 | 0.042 | | 0.252 | 0.867 |  |
| Offset of Tmax | Canopy cover | | | -2.042 | | -0.650 | | 45.647 | 0.000 | | 0.258 | 0.738 |  |
|  | Canopy openness | | | -2.036 | | 0.651 | | 58.020 | 0.000 | | 0.347 | 0.651 |  |
|  | Basal area | | | -2.039 | | -0.242 | | 6.679 | 0.010 | | 0.047 | 0.448 |  |
|  | Crown area | | | -2.037 | | -0.292 | | 5.934 | 0.015 | | 0.066 | 0.476 |  |
|  | Tree height | | | -2.043 | | 0.215 | | 4.537 | 0.033 | | 0.036 | 0.450 |  |
|  | Shade casting ability | | | -2.045 | | -0.300 | | 6.894 | 0.009 | | 0.076 | 0.392 |  |
|  | Forest cover | | | -2.039 | | 0.205 | | 2.024 | 0.155 | | 0.030 | 0.499 |  |
|  | Distance to forest edge | | | -2.045 | | -0.159 | | 1.913 | 0.167 | | 0.020 | 0.410 |  |
|  | Northness | | | -2.046 | | -0.183 | | 4.268 | 0.039 | | 0.027 | 0.417 |  |
|  | Slope | | | -2.046 | | 0.150 | | 1.515 | 0.218 | | 0.018 | 0.425 |  |
|  | Elevation | | | -2.038 | | -0.474 | | 5.553 | 0.018 | | 0.166 | 0.455 |  |
|  | Topographic position | | | -2.044 | | -0.001 | | 0.002 | 0.964 | | 0.000 | 0.408 |  |
|  | Distance to coast | | | -2.037 | | -0.469 | | 5.657 | 0.017 | | 0.173 | 0.403 |  |
| Offset of Tmean | Canopy cover | | | -0.493 | | -0.167 | | 17.564 | 0.000 | | 0.148 | 0.495 |  |
|  | Canopy openness | | | -0.492 | | 0.143 | | 15.294 | 0.000 | | 0.121 | 0.4043 |  |
|  | Basal area | | | -0.493 | | -0.031 | | 0.614 | 0.433 | | 0.005 | 0.356 |  |
|  | Crown area | | | -0.493 | | -0.024 | | 0.177 | 0.674 | | 0.003 | 0.363 |  |
|  | Tree height | | | -0.494 | | 0.070 | | 3.175 | 0.075 | | 0.028 | 0.365 |  |
|  | Shade casting ability | | | -0.494 | | -0.086 | | 3.652 | 0.056 | | 0.041 | 0.383 |  |
|  | Forest cover | | | -0.493 | | 0.041 | | 0.633 | 0.426 | | 0.010 | 0.364 |  |
|  | Distance to forest edge | | | -0.494 | | -0.021 | | 0.204 | 0.652 | | 0.002 | 0.343 |  |
|  | Northness | | | -0.495 | | -0.061 | | 3.040 | 0.081 | | 0.021 | 0.352 |  |
|  | Slope | | | -0.495 | | 0.065 | | 2.037 | 0.153 | | 0.025 | 0.314 |  |
|  | Elevation | | | -0.495 | | 0.120 | | 2.697 | 0.101 | | 0.079 | 0.365 |  |
|  | Topographic position | | | -0.495 | | 0.195 | | 15.738 | 0.000 | | 0.225 | 0.390 |  |
|  | Distance to coast | | | -0.495 | | 0.072 | | 0.862 | 0.353 | | 0.028 | 0.366 |  |

**Table S9**. Contiuned.

| **Autumn** | | | | | | | |  | |  |  |  |  |
| --- | --- | --- | --- | --- | --- | --- | --- | --- | --- | --- | --- | --- | --- |
| Dependent variable | Predictor variable | Intercept | Estimate | Chisq | p | R2m | R2c | |  |  |  |  |  |
| Offset of Tmin | Canopy cover | 0.832 | 0.080 | 2.858 | 0.091 | 0.009 | 0.817 | |  |  |  |  |  |
|  | Canopy openness | 0.832 | -0.131 | 9.751 | 0.002 | 0.024 | 0.823 | |  |  |  |  |  |
|  | Basal area | 0.830 | 0.075 | 3.336 | 0.068 | 0.008 | 0.806 | |  |  |  |  |  |
|  | Crown area | 0.830 | 0.084 | 2.346 | 0.126 | 0.010 | 0.806 | |  |  |  |  |  |
|  | Tree height | 0.831 | -0.010 | 0.044 | 0.834 | 0.000 | 0.803 | |  |  |  |  |  |
|  | Shade casting ability | 0.831 | -0.045 | 0.635 | 0.426 | 0.003 | 0.816 | |  |  |  |  |  |
|  | Forest cover | 0.831 | -0.010 | 0.023 | 0.880 | 0.000 | 0.805 | |  |  |  |  |  |
|  | Distance to forest edge | 0.833 | 0.075 | 1.989 | 0.158 | 0.008 | 0.802 | |  |  |  |  |  |
|  | Northness | 0.832 | 0.014 | 0.117 | 0.732 | 0.000 | 0.803 | |  |  |  |  |  |
|  | Slope | 0.831 | 0.015 | 0.083 | 0.773 | 0.000 | 0.802 | |  |  |  |  |  |
|  | Elevation | 0.817 | 0.764 | 18.726 | 0.000 | 0.479 | 0.900 | |  |  |  |  |  |
|  | Topographic position | 0.828 | 0.268 | 16.839 | 0.000 | 0.126 | 0.788 | |  |  |  |  |  |
|  | Distance to coast | 0.822 | 0.425 | 4.493 | 0.034 | 0.244 | 0.799 | |  |  |  |  |  |
| Offset of Tmax | Canopy cover | -0.934 | -0.200 | 14.271 | 0.000 | 0.097 | 0.621 | |  |  |  |  |  |
|  | Canopy openness | -0.934 | 0.255 | 31.563 | 0.000 | 0.196 | 0.605 | |  |  |  |  |  |
|  | Basal area | -0.932 | -0.077 | 2.567 | 0.109 | 0.016 | 0.490 | |  |  |  |  |  |
|  | Crown area | -0.931 | -0.084 | 1.815 | 0.178 | 0.019 | 0.504 | |  |  |  |  |  |
|  | Tree height | -0.931 | 0.147 | 8.394 | 0.004 | 0.058 | 0.540 | |  |  |  |  |  |
|  | Shade casting ability | -0.935 | -0.125 | 4.282 | 0.039 | 0.047 | 0.426 | |  |  |  |  |  |
|  | Forest cover | -0.930 | 0.134 | 3.849 | 0.050 | 0.045 | 0.561 | |  |  |  |  |  |
|  | Distance to forest edge | -0.933 | -0.045 | 0.586 | 0.444 | 0.006 | 0.476 | |  |  |  |  |  |
|  | Northness | -0.936 | -0.172 | 15.213 | 0.000 | 0.080 | 0.565 | |  |  |  |  |  |
|  | Slope | -0.934 | 0.058 | 0.878 | 0.349 | 0.009 | 0.486 | |  |  |  |  |  |
|  | Elevation | -0.930 | -0.166 | 1.624 | 0.202 | 0.066 | 0.536 | |  |  |  |  |  |
|  | Topographic position | -0.933 | -0.026 | 0.149 | 0.699 | 0.002 | 0.475 | |  |  |  |  |  |
|  | Distance to coast | -0.931 | -0.108 | 0.744 | 0.388 | 0.029 | 0.500 | |  |  |  |  |  |
| Offset of Tmean | Canopy cover | -0.029 | -0.015 | 0.391 | 0.532 | 0.003 | 0.581 | |  |  |  |  |  |
|  | Canopy openness | -0.029 | -0.004 | 0.0.32 | 0.856 | 0.000 | 0.578 | |  |  |  |  |  |
|  | Basal area | -0.029 | 0.019 | 0.780 | 0.377 | 0.004 | 0.572 | |  |  |  |  |  |
|  | Crown area | -0.029 | 0.016 | 0.352 | 0.553 | 0.003 | 0.569 | |  |  |  |  |  |
|  | Tree height | -0.028 | 0.040 | 2.997 | 0.083 | 0.018 | 0.595 | |  |  |  |  |  |
|  | Shade casting ability | -0.029 | -0.037 | 1.704 | 0.192 | 0.014 | 0.610 | |  |  |  |  |  |
|  | Forest cover | -0.028 | 0.023 | 0.619 | 0.431 | 0.006 | 0.568 | |  |  |  |  |  |
|  | Distance to forest edge | -0.028 | 0.016 | 0.358 | 0.550 | 0.003 | 0.576 | |  |  |  |  |  |
|  | Northness | -0.029 | -0.050 | 6.074 | 0.014 | 0.027 | 0.610 | |  |  |  |  |  |
|  | Slope | -0.029 | 0.027 | 0.950 | 0.330 | 0.008 | 0.561 | |  |  |  |  |  |
|  | Elevation | -0.032 | 0.205 | 16.757 | 0.000 | 0.431 | 0.615 | |  |  |  |  |  |
|  | Topographic position | -0.030 | 0.123 | 14.592 | 0.000 | 0.171 | 0.628 | |  |  |  |  |  |
|  | Distance to coast | -0.031 | 0.217 | 23.884 | 0.000 | 0.525 | 0.552 | |  |  |  |  |  |

**Table S9**. Contiuned.

| **Winter** | | |  | | | |  | | |  | |  |  |  |
| --- | --- | --- | --- | --- | --- | --- | --- | --- | --- | --- | --- | --- | --- | --- |
| Dependent variable | Predictor variable | Intercept | | Estimate | Chisq | p | | R2m | R2c | |  |  |  |  |
| Offset of Tmin | Canopy cover | 0.400 | | 0.045 | 1.593 | 0.207 | | 0.004 | 0.828 | |  |  |  |  |
|  | Canopy openness | 0.400 | | -0.072 | 5.236 | 0.022 | | 0.012 | 0.823 | |  |  |  |  |
|  | Basal area | 0.398 | | 0.063 | 4.109 | 0.043 | | 0.009 | 0.819 | |  |  |  |  |
|  | Crown area | 0.397 | | 0.084 | 4.117 | 0.042 | | 0.016 | 0.819 | |  |  |  |  |
|  | Tree height | 0.399 | | 0.000 | 0.000 | 0.997 | | 0.000 | 0.818 | |  |  |  |  |
|  | Shade casting ability | 0.399 | | -0.013 | 0.082 | 0.774 | | 0.000 | 0.822 | |  |  |  |  |
|  | Forest cover | 0.399 | | 0.013 | 0.092 | 0.762 | | 0.000 | 0.818 | |  |  |  |  |
|  | Distance to forest edge | 0.399 | | 0.049 | 1.504 | 0.220 | | 0.005 | 0.820 | |  |  |  |  |
|  | Northness | 0.399 | | -0.020 | 0.479 | 0.489 | | 0.001 | 0.819 | |  |  |  |  |
|  | Slope | 0.398 | | 0.039 | 0.884 | 0.347 | | 0.004 | 0.815 | |  |  |  |  |
|  | Elevation | 0.391 | | 0.514 | 15.779 | 0.000 | | 0.454 | 0.879 | |  |  |  |  |
|  | Topographic position | 0.400 | | 0.235 | 24.579 | 0.000 | | 0.138 | 0.839 | |  |  |  |  |
|  | Distance to coast | 0.389 | | 0.413 | 7.545 | 0.006 | | 0.388 | 0.821 | |  |  |  |  |
| Offset of Tmax | Canopy cover | -0.300 | | -0.025 | 0.309 | 0.578 | | 0.002 | 0.631 | |  |  |  |  |
|  | Canopy openness | -0.301 | | 0.069 | 3.345 | 0.067 | | 0.016 | 0.637 | |  |  |  |  |
|  | Basal area | -0.299 | | -0.032 | 0.701 | 0.402 | | 0.003 | 0.624 | |  |  |  |  |
|  | Crown area | -0.300 | | 0.014 | 0.079 | 0.779 | | 0.001 | 0.623 | |  |  |  |  |
|  | Tree height | -0.300 | | 0.083 | 4.388 | 0.036 | | 0.022 | 0.634 | |  |  |  |  |
|  | Shade casting ability | -0.300 | | -0.036 | 0.622 | 0.430 | | 0.004 | 0.602 | |  |  |  |  |
|  | Forest cover | -0.300 | | 0.028 | 0.248 | 0.619 | | 0.002 | 0.630 | |  |  |  |  |
|  | Distance to forest edge | -0.300 | | -0.032 | 0.477 | 0.490 | | 0.003 | 0.619 | |  |  |  |  |
|  | Northness | -0.300 | | -0.148 | 19.583 | 0.000 | | 0.071 | 0.693 | |  |  |  |  |
|  | Slope | -0.301 | | 0.073 | 2.216 | 0.137 | | 0.018 | 0.615 | |  |  |  |  |
|  | Elevation | -0.298 | | -0.114 | 0.831 | 0.362 | | 0.039 | 0.655 | |  |  |  |  |
|  | Topographic position | -0.299 | | 0.073 | 1.414 | 0.234 | | 0.016 | 0.657 | |  |  |  |  |
|  | Distance to coast | -0.298 | | -0.085 | 0.475 | 0.491 | | 0.023 | 0.641 | |  |  |  |  |
| Offset of Tmean | Canopy cover | -0.026 | | 0.011 | 0.187 | 0.665 | | 0.001 | 0.544 | |  |  |  |  |
|  | Canopy openness | -0.026 | | -0.013 | 0.321 | 0.571 | | 0.002 | 0.541 | |  |  |  |  |
|  | Basal area | -0.026 | | 0.015 | 0.425 | 0.514 | | 0.002 | 0.534 | |  |  |  |  |
|  | Crown area | -0.027 | | 0.043 | 2.163 | 0.141 | | 0.020 | 0.529 | |  |  |  |  |
|  | Tree height | -0.026 | | 0.035 | 1.980 | 0.159 | | 0.012 | 0.547 | |  |  |  |  |
|  | Shade casting ability | -0.026 | | -0.003 | 0.015 | 0.901 | | 0.000 | 0.546 | |  |  |  |  |
|  | Forest cover | -0.026 | | 0.004 | 0.014 | 0.906 | | 0.000 | 0.547 | |  |  |  |  |
|  | Distance to forest edge | -0.026 | | 0.003 | 0.008 | 0.929 | | 0.000 | 0.546 | |  |  |  |  |
|  | Northness | -0.026 | | -0.066 | 9.584 | 0.002 | | 0.044 | 0.590 | |  |  |  |  |
|  | Slope | -0.026 | | 0.045 | 2.280 | 0.131 | | 0.022 | 0.515 | |  |  |  |  |
|  | Elevation | -0.028 | | 0.134 | 4.367 | 0.037 | | 0.167 | 0.604 | |  |  |  |  |
|  | Topographic position | -0.025 | | 0.137 | 14.877 | 0.000 | | 0.147 | 0.708 | |  |  |  |  |
|  | Distance to coast | -0.029 | | 0.145 | 5.215 | 0.022 | | 0.209 | 0.568 | |  |  |  |  |

**Table S9**. Contiuned.

| **All year** |  |  | |  | | |  | | |  |  |  |
| --- | --- | --- | --- | --- | --- | --- | --- | --- | --- | --- | --- | --- |
| Dependent variable | Predictor variable | Intercept | Estimate | | Chisq | p | | R2m | R2c |  |  |  |
| Offset of Tmin | Canopy cover | 0.863 | 0.076 | | 2.736 | 0.098 | | 0.007 | 0.851 |  |  |  |
|  | Canopy openness | 0.864 | -0.126 | | 9.685 | 0.002 | | 0.019 | 0.857 |  |  |  |
|  | Basal area | 0.860 | 0.076 | | 3.546 | 0.060 | | 0.007 | 0.843 |  |  |  |
|  | Crown area | 0.860 | 0.081 | | 2.268 | 0.132 | | 0.008 | 0.842 |  |  |  |
|  | Tree height | 0.862 | -0.014 | | 0.101 | 0.750 | | 0.000 | 0.841 |  |  |  |
|  | Shade casting ability | 0.861 | -0.009 | | 0.020 | 0.887 | | 0.000 | 0.843 |  |  |  |
|  | Forest cover | 0.862 | 0.012 | | 0.048 | 0.826 | | 0.000 | 0.840 |  |  |  |
|  | Distance to forest edge | 0.863 | 0.089 | | 2.983 | 0.084 | | 0.009 | 0.841 |  |  |  |
|  | Northness | 0.861 | -0.004 | | 0.013 | 0.910 | | 0.000 | 0.841 |  |  |  |
|  | Slope | 0.861 | 0.027 | | 0.252 | 0.616 | | 0.001 | 0.839 |  |  |  |
|  | Elevation | 0.847 | 0.943 | | 25.394 | 0.000 | | 0.549 | 0.936 |  |  |  |
|  | Topographic position | 0.863 | 0.336 | | 30.229 | 0.000 | | 0.168 | 0.845 |  |  |  |
|  | Distance to coast | 0.851 | 0.464 | | 4.335 | 0.037 | | 0.258 | 0.839 |  |  |  |
| Offset of Tmax | Canopy cover | -0.797 | -0.315 | | 24.039 | 0.000 | | 0.133 | 0.704 |  |  |  |
|  | Canopy openness | -0.798 | 0.328 | | 34.466 | 0.000 | | 0.177 | 0.666 |  |  |  |
|  | Basal area | -0.789 | -0.136 | | 5.262 | 0.022 | | 0.031 | 0.529 |  |  |  |
|  | Crown area | -0.788 | -0.140 | | 3.342 | 0.068 | | 0.032 | 0.525 |  |  |  |
|  | Tree height | -0.792 | 0.156 | | 6.065 | 0.014 | | 0.039 | 0.549 |  |  |  |
|  | Shade casting ability | -0.794 | -0.188 | | 6.402 | 0.011 | | 0.064 | 0.472 |  |  |  |
|  | Forest cover | -0.789 | 0.138 | | 2.518 | 0.113 | | 0.028 | 0.574 |  |  |  |
|  | Distance to forest edge | -0.792 | -0.101 | | 1.855 | 0.173 | | 0.017 | 0.514 |  |  |  |
|  | Northness | -0.792 | -0.195 | | 12.694 | 0.000 | | 0.062 | 0.567 |  |  |  |
|  | Slope | -0.792 | 0.144 | | 3.408 | 0.065 | | 0.033 | 0.535 |  |  |  |
|  | Elevation | -0.785 | -0.387 | | 5.784 | 0.016 | | 0.208 | 0.605 |  |  |  |
|  | Topographic position | -0.791 | -0.034 | | 0.167 | 0.683 | | 0.002 | 0.505 |  |  |  |
|  | Distance to coast | -0.784 | -0.304 | | 3.793 | 0.051 | | 0.152 | 0.515 |  |  |  |
| Offset of Tmean | Canopy cover | -0.070 | -0.056 | | 4.333 | 0.037 | | 0.040 | 0.386 |  |  |  |
|  | Canopy openness | -0.070 | 0.040 | | 2.611 | 0.106 | | 0.021 | 0.355 |  |  |  |
|  | Basal area | -0.069 | 0.003 | | 0.029 | 0.865 | | 0.000 | 0.328 |  |  |  |
|  | Crown area | -0.069 | 0.013 | | 0.247 | 0.619 | | 0.002 | 0.320 |  |  |  |
|  | Tree height | -0.069 | 0.045 | | 3.053 | 0.081 | | 0.026 | 0.344 |  |  |  |
|  | Shade casting ability | -0.069 | -0.043 | | 1.952 | 0.162 | | 0.023 | 0.379 |  |  |  |
|  | Forest cover | -0.069 | 0.031 | | 0.990 | 0.320 | | 0.013 | 0.334 |  |  |  |
|  | Distance to forest edge | -0.069 | 0.001 | | 0.005 | 0.946 | | 0.000 | 0.334 |  |  |  |
|  | Northness | -0.069 | -0.073 | | 10.424 | 0.001 | | 0.069 | 0.408 |  |  |  |
|  | Slope | -0.070 | 0.072 | | 5.487 | 0.019 | | 0.072 | 0.293 |  |  |  |
|  | Elevation | -0.071 | 0.137 | | 11.753 | 0.001 | | 0.249 | 0.351 |  |  |  |
|  | Topographic position | -0.068 | 0.151 | | 22.519 | 0.000 | | 0.291 | 0.459 |  |  |  |
|  | Distance to coast | -0.072 | 0.125 | | 8.747 | 0.003 | | 0.209 | 0.337 |  |  |  |

## Appendix S10 | Correlation matrix and histograms of predictor variables


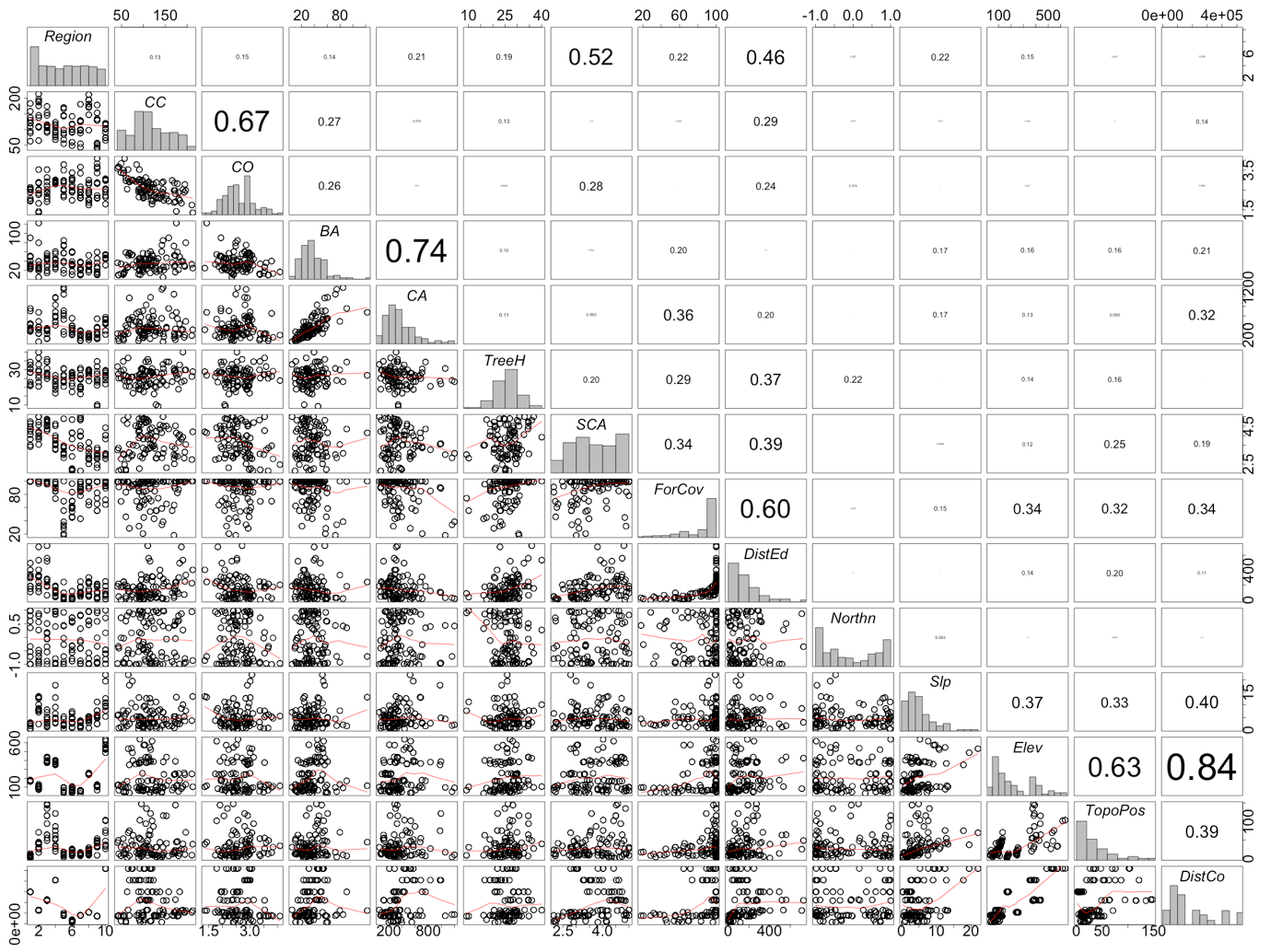


**Figure S10**. Matrix showing scatterplots, histograms and Pearson correlation coefficients for the explanatory variables tested. CC: canopy cover; CO: canopy openness (log-transformed); BA: basal area; TreeH: tree height; SCA: shade casting ability; ForCor: forest cover; DistEd: distance to forest edge; Northn: topographic northness; Slp: topographic slope; Elev: elevation; TopoPos; topographic position; DistCo: distance to coast (see Table 1 in the main text for further details).

## Appendix S11 | Predictive performance of canopy variables for Tmax offset

**Table S11**. Predictive model performance of canopy variables explaining the offset of Tmax during summer, as determined by cross-validation (see main text for details). We report the *R^2^* value based on the pearson’s correlation coefficient between the predicted and observed values, as well as their Root Mean Squared Error (RMSE). Predictions were made for each predictor variable combined with *distance to coast*, i.e., the variable with a relatively large influence on the buffering of maximum temperatures during spring and summer (Table S8). For comparison of linearity vs. non-linearity we used linear mixed-effects models (LMM) and the general additive mixed-effects models (GAMM). We did not investigate the predictive performance of canopy variables for Tmin offset because of the weak statistical relationship between the two.

| **Season** | **Dependent variable** | **Predictor variable** | **CVR^2^ lmm** | **RMSE lmm** | **CVR^2^ gamm** | **RMSE gamm** |
| --- | --- | --- | --- | --- | --- | --- |
| Summer | Offset of Tmax | Canopy cover | 0.24 | 1.01 | 0.33 | 0.92 |
|  |  | Canopy openness | 0.43 | 0.83 | 0.24 | 0.99 |
|  |  | Basal area | 0.10 | 1.06 | 0.09 | 0.07 |
|  |  | Crown area | 0.08 | 1.09 | 0.08 | 1.10 |
|  |  | Tree height | 0.09 | 1.06 | 0.06 | 1.10 |
|  |  | Shade casting ability | 0.17 | 1.01 | 0.20 | 0.99 |

**References**

Abatzoglou J. T., Dobrowski S. Z., Parks S. A. & Hegewisch K. C. (2018). TerraClimate, a high-resolution global dataset of monthly climate and climatic water balance from 1958–2015. *Scientific Data*, *5*, 170191.

Campbell G. S. (1986). Extinction coefficients for radiation in plant canopies calculated using an ellipsoidal inclination angle distribution. *Agricultural and Forest Meteorology*, *36*, 317–321.

Fischer H. S. (2015). On the combination of species cover values from different vegetation layers. *Applied Vegetation Science*, *18*(1), 169–170. https://doi.org/10.1111/avsc.12130.

Jucker T., Caspersen J., Chave J., Antin C., Barbier N., Bongers F., … Coomes D. A. (2016). Allometric equations for integrating remote sensing imagery into forest monitoring programmes. *Global Change Biology*. https://doi.org/10.1111/gcb.13388.

Karger D. N., Conrad O., Böhner J., Kawohl T., Kreft H., Soria-Auza R. W., … Kessler M. (2017). Climatologies at high resolution for the earth’s land surface areas. *Scientific Data*, *4*, 170122. https://doi.org/10.1038/sdata.2017.122.

Kollas C., Randin C. F., Vitasse Y. & Körner C. (2014). How accurately can minimum temperatures at the cold limits of tree species be extrapolated from weather station data? *Agricultural and Forest Meteorology*, *184*, 257–266. https://doi.org/https://doi.org/10.1016/j.agrformet.2013.10.001.

Rolland C. (2003). Spatial and Seasonal Variations of Air Temperature Lapse Rates in Alpine Regions. *Journal of Climate*, *16*, 1032–1046.
